# mRNA-Based Cancer Vaccines: A Therapeutic Strategy for the Treatment of Melanoma Patients

**DOI:** 10.3390/vaccines9101060

**Published:** 2021-09-23

**Authors:** Maryam Bidram, Yue Zhao, Natalia G. Shebardina, Alexey V. Baldin, Alexandr V. Bazhin, Mohamad Reza Ganjalikhany, Andrey A. Zamyatnin, Mazdak Ganjalikhani-hakemi

**Affiliations:** 1 Department of Cell and Molecular Biology, Faculty of Biological Science and Technology, University of Isfahan, Isfahan 8174673441, Iran; m.bidram@sci.ui.ac.ir (M.B.); m.ganjalikhany@sci.ui.ac.ir (M.R.G.); 2 Department of General, Visceral and Transplant Surgery, Ludwig-Maximilians University of Munich, 81377 Munich, Germany; yue.zhao@med.uni-muenchen.de (Y.Z.); alexandr.bazhin@med.uni-muenchen.de (A.V.B.); 3 Institute of Molecular Medicine, Sechenov First Moscow State Medical University, 119991 Moscow, Russia; natuskasheb@gmail.com; 4 Belozersky Institute of Physico-Chemical Biology, Lomonosov Moscow State University, 119992 Moscow, Russia; alexey-baldin@belozersky.msu.ru; 5 V.I. Kulakov National Medical Research Center of Obstetrics, Gynecology and Perinatology, 117997 Moscow, Russia; 6 German Cancer Consortium (DKTK), Partner Site Munich, 81377 Munich, Germany; 7 Department of Biotechnology, Sirius University of Science and Technology, 1 Olympic Ave, 354340 Sochi, Russia; 8 Faculty of Health and Medical Sciences, University of Surrey, Guildford GU2 7X, UK; 9 Department of Immunology, Faculty of Medicine, Isfahan University of Medical Sciences, Isfahan 8174673441, Iran

**Keywords:** melanoma cancer, mRNA vaccine, therapeutic, delivery systems, immune checkpoint

## Abstract

Malignant melanoma is one of the most aggressive forms of cancer and the leading cause of death from skin tumors. Given the increased incidence of melanoma diagnoses in recent years, it is essential to develop effective treatments to control this disease. In this regard, the use of cancer vaccines to enhance cell-mediated immunity is considered to be one of the most modern immunotherapy options for cancer treatment. The most recent cancer vaccine options are mRNA vaccines, with a focus on their usage as modern treatments. Advantages of mRNA cancer vaccines include their rapid production and low manufacturing costs. mRNA-based vaccines are also able to induce both humoral and cellular immune responses. In addition to the many advantages of mRNA vaccines for the treatment of cancer, their use is associated with a number of challenges. For this reason, before mRNA vaccines can be used for the treatment of cancer, comprehensive information about them is required and a large number of trials need to be conducted. Here, we reviewed the general features of mRNA vaccines, including their basis, stabilization, and delivery methods. We also covered clinical trials involving the use of mRNA vaccines in melanoma cancer and the challenges involved with this type of treatment. This review also emphasized the combination of treatment with mRNA vaccines with the use of immune-checkpoint blockers to enhance cell-mediated immunity.

## 1. Introduction

Melanoma is a malignant tumor that originates from melanocytes. It is one of the most aggressive forms of skin cancer and has a low survival rate. In the United States, in 2021, the estimated number of new cases has already reached 106,000, with the number of deaths being about 7000, which are constantly growing [1]. Although advances in melanoma diagnosis have improved the chance of early detection, the prognosis for patients with advanced or metastatic melanoma remains poor. One of the most threatening properties of malignant melanoma is its ability to metastasize rapidly. To predict the likelihood of metastasis, the following factors are assessed: the depth of invasion of the primary tumor, the presence of an ulcer, micrometastases in regional lymph nodes, and the number of mitoses in thin tumors [2]. The stage of malignancy in melanoma patients determines the type of treatment required [3]. Nowadays, there are several therapies available, including chemotherapy, radiation therapy, immunotherapy, and surgery. Of these, immunotherapy is the most modern method and is continuing to evolve [4]. Ipilimumab (anti-CTLA-4 monoclonal antibody), nivolumab, and pembrolizumab (anti-PD-1 monoclonal antibodies) were the first immunotherapies approved by the US Food and Drug Administration (FDA) for the treatment of metastatic cutaneous melanomas [5,6]. These immunotherapies represent checkpoint inhibitors to enhance the immune system. Combination therapy with ipilimumab and nivolumab was also approved in 2015 for the treatment of stage III and IV melanoma patients [7]. Immunotherapy is considered a standard treatment option for advanced melanoma.

Conventional treatments such as chemotherapy do not specifically target tumor cells, so they also damage the normal dividing cells. Additionally, the use of suboptimal doses of cancer chemotherapeutic agents to reduce side effects can lead to treatment failure. It has been found that oncological diseases are capable of progressing only when the adequate functioning of the immune system is disrupted, as this ensures the control over oncogenic viruses and abnormal cells. Accordingly, active studies on cancer immunotherapies that suppress tumor development continue. Immunotherapies can be differentiated in terms of their activity and specificity. As an active therapy type, cytokines or synthetic molecules are usually used. To activate a specific immune response, vaccines based on tumor or viral antigens are used. The main type of passive, nonspecific therapy is adoptive cell therapy, in which effector cells are activated outside the body and then injected back into the patient. With the help of tumor-specific monoclonal antibodies, a passive-specific immune response can be achieved [4,8,9].

Unlike vaccines against infectious diseases, cancer vaccines are focused on the treatment of the disease rather than on its prevention. The aim of therapeutic cancer vaccines is to induce specific stimulation of the patient’s immune system using tumor antigens to ultimately trigger an antitumor response, leading to tumor removal. There are several types of cancer vaccines: (1) whole-cell vaccines, which are subdivided into autologous, modified and unmodified, and allogeneic; (2) vaccines based on heat shock proteins, gangliosides, or peptides; (3) vaccines based on dendritic cells; (4) vaccines based on recombinant viruses; and (5) vaccines based on DNA or mRNA [10]. A potential target for cancer vaccines is somatic point mutations in tumors that trigger or control the neoplastic process. Melanoma, thus, represents an excellent target for cancer vaccine treatment since it is a malignant tumor with one of the highest mutation prevalence, namely a high tumor mutation burden (TMB) [11]. Such a feature of melanoma makes it highly immunogenic, providing a lot of antigens to choose from for vaccine formulation. Moreover, tumors with high TMB are characterized by tumor microenvironment highly infiltrated by lymphocytes. Such tumors, including melanoma, in particular, have the highest response rates to checkpoint inhibitors [12,13].

The most recently developed cancer vaccine options are mRNA vaccines. Initially, for cancer immunotherapy, mRNA was used only as a template encoding tumor-associated antigens, but due to its versatility and design variability, the therapeutic potential of mRNA is now considered limitless. The simplicity of mRNA vaccines greatly reduces complications generally associated with the production of biological vaccines, such as the handling of infectious agents and genetic variability or environmental risks. mRNA-based vaccines can be produced easily and rapidly. A major reason for the use of mRNA vaccines is their superior safety profile compared with those of pDNA and viral vectors. mRNA represents the minimal genetic vector and contains only the elements directly required for the expression of the encoded protein [14]. mRNA can now be used for the following purposes: (1) to deliver tumor-specific monoclonal antibodies, monoclonal antibodies that block immune checkpoints, and their fragments to create bispecific antibodies or chimeric antigen receptors; (2) to deliver toxic proteins that induce the death of cancer cells; (3) to modulate tumor-associated dendritic cells; (4) to modulate the suppressive tumor microenvironment; (5) to modulate the cytokines in the tumor microenvironment; (6) and to generate cancer T cells. These mRNA applications were reviewed in detail by Hoecke et al. [15].

## 2. Melanoma Antigens

As melanoma cells progress, they express intracellular or cell-surface molecules, which can be both tumor-specific and ectopic normal proteins. An ideal immunotherapy target would be an antigen that is immunogenic and specific to cancer cells. The identification and characterization of such antigens can contribute to gaining a better understanding of tumor progression and differentiation, and they could also be regarded as targets for immunotherapies. Different melanoma-associated antigens are characterized by their unique compositions, cellular locations, and the stages at which they begin to be expressed [16]. Depending on their characteristics, the following melanoma antigens can be distinguished: antigens associated with melanocyte differentiation, oncofetal or cancer-germline antigens, and cell membrane proteins [17].

Melanocyte differentiation antigens include tyrosinase, tyrosinase-related proteins (TRP-1 and TRP-2), melanocyte antigen (MELAN-A/MART-1), glycoprotein 75 (gp75), and glycoprotein 100 (gp100) [16,18,19]. Melanocyte differentiation antigens are not mutant; however, they are only expressed in melanoma cells and melanocytes at different stages of differentiation [20]. These antigens are localized in compartments where melanin is synthesized, that is, in the corresponding organelles and melanosomes, and they act as highly specific markers of melanocyte differentiation [21,22]. Thanks to tumor-infiltrating lymphocytes, which can detect antigens associated with melanocyte differentiation, it has become possible to use such antigens as targets for immunotherapy [23].

Cancer-germline antigens include more than 80 proteins, which are combined into families. These are the melanoma-associated antigen family (MAGE-family), the BM antigen family (BAGE-family), the G-antigen family (GAGE-family), the synovial sarcoma family of the X chromosome breakpoint (SSX-family), and NY-ESO-1, which has no homologs. Normally, cancer-germline antigens are expressed in the placenta, trophoblasts, medullary epithelial cells of the thymus, and germ cells of the testes at various stages of spermatogenesis [24,25]. The expression of some types of antigens has been observed in somatic tissues, mainly at the developmental stage [24,25]. The expression of this group of antigens is generally limited to germ cells, so their appearance in adults can be associated with the occurrence of various types of cancer, including melanoma.

Membrane-associated proteins are considered targets for immunotherapies, since their expression is increased in melanoma cancer cells [26]. At this stage, the most commonly studied melanoma receptors include integrins, melanoma chondroitin sulfate proteoglycan (MCSP), immunoglobulin superfamily molecules, melanotransferrin (MTf), and S100. In a review by Pitcovski et al., different types of membrane-associated antigens are considered in more detail [17].

Melanoma-associated antigens are considered targets for CAR therapy. For example, CAR-lymphocytes that recognize NY-ESO have been associated with a strong immune response [27]. CAR-lymphocytes, which recognize melanoma antigens such as MART-1 and gp100, have been shown to cause an autoimmune reaction that affects healthy melanocytes of the skin, eyes, and other tissues, resulting in side effects in the form of vitiligo and the impairment of vision and hearing [28]. Peptide vaccines against melanoma are most often based on gp100, MAGE, Melan A (MART-1), and NY-ESO. For example, after the introduction of a vaccine based on gp100 HLA-A2, positive patients with stage I–c melanoma displayed a specific cytotoxic immune response involving the formation of a pool of memory T cells [29].

In addition to the tumor-associated antigens (TAAs) mentioned above, tumor-specific antigens (TSAs) are also considered an important target for immunotherapies. Tumor-specific antigens, known as neoantigens, are new proteins caused by mutations that occur in tumor DNA. In fact, these mutational antigens are specifically expressed in tumor cells and are not present in normal cells. Neoepitopes can be divided into two classes: neoantigens that are only found in a particular type of cancer are called shared, and those that are specific to a patient are called personalized [30]. BRAF and NRAS mutations, for example, are shared neoepitopes that are observed in approximately 50% and 15–25% of melanomas, respectively [31,32]. These characteristics suggest that neoantigen cancer vaccines could be a promising prospect for cancer immunotherapy, as studies show that these vaccines may elicit stronger immune responses than TAA-loaded DC vaccines [33]. For vaccination involving neoantigens, the whole genome is sequenced after tumor biopsy and compared with healthy tissues to identify existing mutations. Each mutation is then evaluated using bioinformatics algorithms to determine its affinity to bind to the MHC molecule and elicit an immune response [34,35].

The analysis of whole-genome sequences from major melanoma subtypes, including cutaneous, acral (hands and feet), and mucosal subtypes, has demonstrated a different genomic landscape. A lower TMB has been observed for acral and mucosal melanomas than for cutaneous melanoma. The heavily mutated landscape of cutaneous melanoma, which results from coding and non-coding mutations, is attributed to its underlying mechanism of ultraviolet radiation-induced DNA damage. In contrast, mucosal and acral melanomas are dominated by structural variants that are not attributed to ultraviolet radiation. Therefore, these subtypes differ in terms of their pathogenesis and therapeutic targets (detailed by Hayward, N.K. et al.) [36].

The combination of melanoma antigens can be used for diagnosis and determination of the disease stage and prognosis, which is certainly important for determining treatment tactics and developing a timely response to pathology. Knowledge about the composition, cellular location, and stage at which antigens begin to be expressed may assist with the development of melanoma immunotherapies based on antigen-specific immunization. At the same time, it is necessary to take into account the limitations associated with the characteristic properties of each antigen group. One of the progressive directions in the melanoma immunotherapies is the development of personalized vaccines.

## 3. mRNA Vaccines: General Features

### 3.1. The Basis of mRNA Vaccines

Currently, mRNA-based vaccines are attracting more and more interest in the scientific and medical communities. Against the background of the global COVID-19 pandemic, the issue of developing vaccines for disease prevention has become the focus. One of the most progressive and effective options is the development of mRNA-based vaccines. One of the advantages of mRNA-based vaccines is their ability to induce both humoral and cellular immunity, in particular, through the induction of the CD8^+^ T cell response, which is of great importance in the fight against tumors [37]. At the same time, mRNA vaccines, unlike DNA vaccines, do not have serious side effects such as integration into the patient’s genome. Such integration side effects might include gene disruption, insertional mutagenesis, cell death, and even tumorogenesis [38]. In addition, mRNA functions in the cytoplasm and does not penetrate into the nucleus of the target cell that facilitates delivery. Finally, a significant advantage of mRNA vaccines is their rapid and inexpensive production, allowing high yields of the desired product to be produced under in vitro conditions.

**Figure 1:**
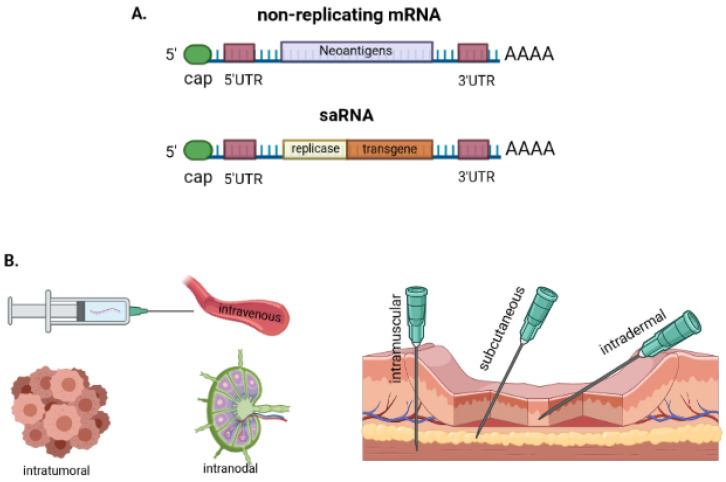
Non-replicating and self-amplifying mRNA (**A**) and administration routes of mRNA vaccines (**B**).

RNA vaccines can be divided into two types: (1) vaccines based on conventional non-replicating mRNA that only encode the antigen of interest, and (2) vaccines based on self-amplifying mRNA (saRNA), which is produced from single-stranded RNA viruses and encodes the viral replication apparatus (Figure 1A). The mechanisms of action of saRNA vaccines have been discussed in detail in previous reviews [39]. Conventional non-replicating mRNA consists of five structural elements: (1) cap structures; (2) a 5′-untranslated region (5′-UTR); (3) an open reading frame (ORF) encoding antigens of interest; (4) a 3′-UTR; and (5) an adenine repeating nucleotide sequence that forms a polyadenine (poly(A)) tail. Unlike saRNA, ordinary mRNA is small due to its simpler structure and the presence of only one ORF.

saRNA encodes not only an antigen-encoding gene, but also a gene responsible for viral RNA replication [40]. saRNA, due to amplification of the RNA template in the target cells, expresses high levels of the gene of interest. Depending on the preparation method, saRNA can be divided into (1) plasmid-based DNA saRNA, (2) virus-like particle delivery saRNA, and (3) in vitro transcribed saRNA [41]. Based on these three types of saRNA, Beissert et al. developed trans-amplifying RNA (taRNA) to activate the immune response, a process that is safe, processable, and easy to optimize [42].

**Figure 2:**
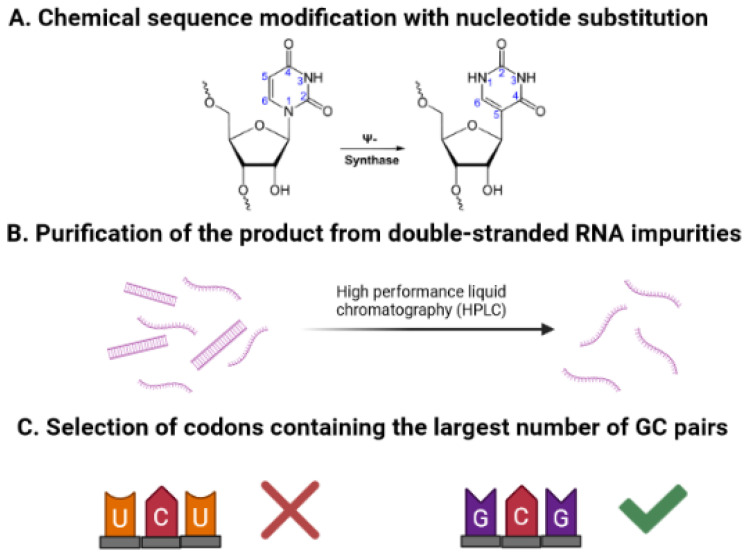
Methods of mRNA nuclease degradation reduction. The chemical modification of uridine to pseudouridine (**A**), purification of the product from double-stranded RNA using high-performance liquid chromatography (HPLC) (**B**), and choice of codons containing more G:C pairs (**C**) are performed to reduce the sensitivity of mRNA to digestion by nucleases.

mRNA vaccines are associated with a set of problems that need to be considered. First, these vaccines are highly sensitive to nuclease degradation and immunogenicity. Over the past decade, a wide variety of options have been explored to overcome these obstacles (Figure 2). The aim was to obtain an immunologically quiet mRNA product, since the decisive factor associated with this drawback is the rapid recognition of mRNA molecules by innate immunity sensors. The uridine-rich sequence is believed to be a key factor in the activation of Toll-like receptors [43]. Nelson et al. considered and demonstrated a variant containing N1-methyl-pseudouridine (1mΨ)-modified mRNA with the removal of double-stranded RNA (dsRNA) impurities [44] (Figure 2A,B). Their study confirmed that replacing uridine with 1mΨ in mRNA changes its interaction with pattern recognition receptors (PRR), suppresses the stimulation of innate immunity, and increases the stability of the molecule. In addition, the combination of this technique with purification of the product to reduce dsRNA impurities, which are formed during transcription and also affect innate immune activity, contributes to the sequence of interest having the most favorable level of expression. This is due to the fact that mammalian Toll-like receptor 3 (TLR3) recognizes dsRNA and induces activation of NF-κB [45]. Accordingly, the purification of the product from dsRNA contributes to a decrease in immunogenicity. Other types of chemical sequence modifications, such as the replacement of cytidine with 5-methylcytidine (m5C); the replacement of uridine with 5-methyluridine (m5U), 2-thiuridine (s2U), 5-methoxyuridine (5moU), or pseudouridine (ψ); and the replacement of adenosine with N1-methyladenosine (m1A) or N6-methyladenosine (m6A) have been investigated [46]. Another way to avoid an innate immune response and to ensure sufficient expression of the required protein might be optimization of the mRNA sequence in combination with purification from dsRNA, as was demonstrated by Thess et al. [47]. In their study, for each amino acid contained in the protein of interest, only the richest guanine and cytosine codons were used. Although the selection of GC-rich mRNA is described as a method to improve mRNA half-life, it is controversial. Indeed, AU-rich elements located in the 3′-UTR can act to destabilize mRNA [48,49,50]. It has been noted that mRNAs that harbor coding region instability elements happen to be GC-poor (i.e., factor VIII, IL2, c-myc, c-fos, HPV, HIV-1 mRNAs). However, it is not known whether a general correlation exists between cellular mRNA lifetime and GC content. Moreover, it has been shown that GC content on mRNA does not significantly affect the cellular mRNA lifetime, while the effect of increased expression level of GC-rich genes was due to their several-fold higher transcription efficacy [51].

Additional methods for stabilizing the mRNA molecule include the use of synthetic analogs of the cap and cap enzymes, regulatory elements in the 5′-UTR and 3′-UTR, and the addition of poly(A) tails that screen mRNA. These methods significantly increase protein translation [52,53,54,55]. Strategies for improving the translation efficiency are discussed in detail in a review by Miao et al. [56].

**Figure 3:**
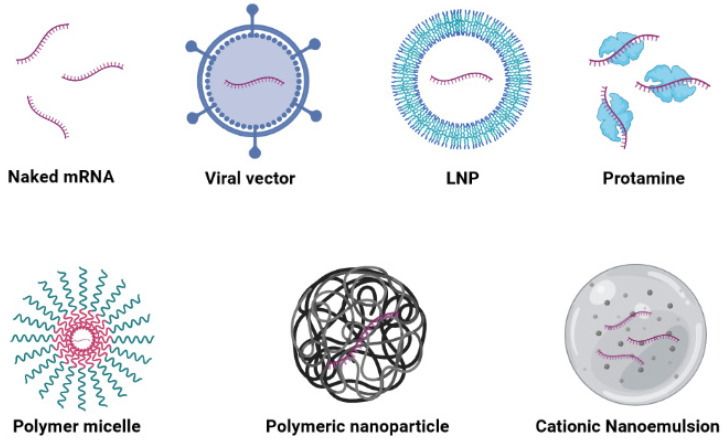
Various carriers for mRNA vaccine delivery.

Another problem associated with mRNA-based vaccines is the delivery of molecules into the cytoplasm. mRNA is a large, hydrophilic, negatively charged molecule that cannot freely enter target cells through the lipid bilayer membrane. In recent years, methods of delivering mRNA to target cells have been actively studied [57] (Figure 3). However, it is known that, under certain conditions, naked mRNA can enter cells and induce an immune response against the encoded antigen.

The choice of mRNA-based vaccine administration method plays an equally important role in determining the quality and intensity of the immune response. Intradermal, subcutaneous, and intramuscular drug administration methods are usually used to inoculate cancer mRNA vaccines (Figure 1B). In a study by Pardi et al., mRNA-lipid nanoparticles (mRNA-LNP) were used to assess the effects of different vaccine administration routes on antigen expression [58]. With intramuscular and subcutaneous injections, expression of the mRNA-LNP protein was higher than with intradermal injection. Intranodal immunization under ultrasound control is also an attractive option due to the high concentration of dendritic cells in the draining lymph nodes [59]. Additionally, intratumoral delivery is a potential method, since it provokes a quick response by local immune cells. In a study by Jeught et al., intratumoral delivery of fusion mRNA was shown to have significant therapeutic potential, which was enhanced by immune checkpoint inhibitors [60].

### 3.2. Vaccine Optimization by Improving mRNA Translation and Stability

Because mRNA is sensitive and vulnerable to environmental enzymes, its stabilization should guarantee protein expression. Making various modifications to the structural elements of in vitro transcribed (IVT) mRNA significantly increases the duration, and hence amount, of encoded protein production. Modifiable elements include the 5′ cap, 5′- and 3′-UTRs, the coding region, and the poly(A) tail [61]. During the transcription process, a 7-methylguanosine (m7G) cap is linked to mRNA by a 5′-5′-triphosphate (ppp) bridge (m7GpppNpNp…), which is attached to eukaryotic translation initiation factor 4E (EIF4E) in the early stages of translation [62]. Therefore, the mRNA capping process is vital for the stability and maturity of mRNA. The use of recombinant vaccinia virus capping enzymes and synthetic cap analogs are two important approaches that can be used to create different versions of the 5′ cap for use in IVT mRNA [53,63]. During this process, the cap may also be inserted in the reverse direction, an issue that can be overcome with the use of anti-reverse cap analogs to increase translation efficiency [54].

Modification of the 5′- and 3′-UTRs is another way to improve the translation and increase the half-life of IVT mRNA. The 5′- and 3′-UTRs contain regulatory sequence elements that are located upstream and downstream of the initiation and termination codons, respectively [64]. Studies have shown that, in orthopoxviruses, mRNA 5′-UTR is involved in the inhibition of both decapping and 3′-5′ exonucleolytic degradation activities [65]. The 3′-UTR of α-globin and β-globin mRNAs has been widely targeted in clinical trials [66]. The 3′-UTR plays a crucial role in gene expression due to containing sequences such as AU-rich elements (AREs) and the poly(A) tail. In some therapeutic applications, limited-length proteins have required the presence of an AU-rich area [67].

The addition of a suitable length poly(A) tail at the 3′ end of mRNA also plays an important role in its successful translation and stability. The poly(A) tail can be added to IVT mRNA either through a template vector or by recombinant poly(A) polymerase after the transcription process has occurred [52,61].

Coding region modification is another way to strengthen mRNA stability and translation. Codon optimization is performed to avoid the occurrence of rare codons, which are replaced with repeatedly used synonymous codons. These codons do not alter the amino acid sequence of the protein, as they have the same cognate and tRNA, but they accelerate the translation process and increase the protein yield [68,69]. However, some proteins require slower translation rates for reasons such as ensuring correct folding, as is the case when rare codons are present [70]. Usually, to evaluate codon optimization, the protein resulting from DNA plasmids that carries the wild-type sequence is compared with the protein with the modified and optimized sequence [71]. Codon-optimization of IVT mRNA has been successfully conducted in studies focused on producing vaccines for viral and non-viral infections [72,73].

### 3.3. Various Carriers for mRNA Vaccine Delivery

Another challenge for mRNA vaccines is their passage through the membrane and delivery to the cells, because mRNA is an unstable, large, negatively charged molecule that has difficulty crossing the membrane structure. Because mRNA cannot pass through the membrane by passive diffusion, RNA-based drugs are usually taken up by endocytosis. Although naked mRNA vaccines can be injected directly into cells, such as dendritic cells, delivery carriers are required for significant rates of expression and inhibition to occur [74]. Therefore, various strategies have been developed to introduce mRNA vaccines to cells, including viral-vector-based delivery, lipid-based delivery, polymer-based delivery, hybrid-carrier-based delivery, and peptide-based delivery (Figure 3). These delivery carriers prevent the degradation of mRNA by RNase enzymes and facilitate its entry into cells and subsequent escape from the endosome, allowing it to reach lymphoid organs and cellular targets to eventually produce the desired antigen and immune response [75].

#### 3.3.1. Naked mRNA Vaccines

Unlike carrier-based mRNA vaccines, naked mRNA delivery is usually realized by direct injection of the mRNA solution. naked mRNA is commonly dissolved in Ringer’s solution or lactated Ringer’s solution for vaccination [76,77]. Ca^2+^ is used as a component of these two solutions, since it is known to improve the uptake of mRNA in an ion-dependent manner [78]. naked mRNA dissolved in Ringer’s solution has already been tested in anticancer clinical trials. For instance, in a study by Sahin et al., naked mRNA was diluted in Ringer’s solution at a concentration of 1.0 mg mL^−1^ and injected into separate inguinal lymph nodes in thirteen melanoma patients [79]. Patients showed good tolerance, and promising results were achieved in this clinical trial.

The cellular uptake mechanism of naked mRNA remains elusive. It is known that naked mRNA molecules cannot penetrate the cell membrane freely. Several scientists hypothesized that the uptake of naked mRNA mainly occurs due to a variety of DC-mediated endocytic pathways [74,80,81,82]. This process ensures the translation of antigen-encoding mRNA and promotes the activation of DC and T cells, leading to the formation of antitumor adaptive immunity. However, to determine the mechanism underlying this process, further investigation is required.

Unlike DNA vaccines, naked mRNA shows superiority in terms of its low level of toxicity due to the bacteria-free manufacturing procedures used for its production [83]. Furthermore, patients who receive naked mRNA injections are free from the threat of genome integration, which occurs in DNA vaccination. Naked mRNA also has the advantage of quicker protein translation than DNA molecules. Instead of complex antigen generation, beginning from DNA transcription to mRNA, followed by translation processes, once naked mRNA molecules reach the cytosol, ribosomes combine with mRNA and launch the translation procedure promptly, resulting in a rapid immune response following naked mRNA administration. Thus, mRNA vaccines usually achieve the targeted immunity of hosts more quickly than DNA vaccination methods. However, the limitations of naked mRNA molecules as vaccines are obvious. The instability of mRNA molecules frequently leads to their degradation by host RNases. Moreover, their low translation efficiency reduces their ability to generate sufficient antigens to trigger an immune response when used as vaccines. Further, unwanted innate immunity against naked mRNA may be stimulated. To overcome these disadvantages, several mRNA structure modifications have been applied, for example, sequence optimization, modification of nucleosides, and purification of mRNA, as mentioned above. Furthermore, following alteration of the administration pathway to avoid having RNase in the bloodstream, for example, by using intranodal, intradermal, and intramuscular administration methods, naked mRNA vaccines have still shown promising results. Notably, Sahin and colleagues administered naked, unmodified mRNA encoding neoantigens in an intranodal manner, and this was shown to bolster robust specific T cell immune responses in melanoma patients, demonstrating that the challenges associated with the use of naked mRNA vaccines in clinical practice are surmountable [79].

#### 3.3.2. Viral Vectors

Delivery systems can be based on viral or non-viral vectors. In vaccines based on viral vectors, modified viruses are used to deliver the genetic code of the antigen to the target cells. These vaccines work similarly to natural infections caused by viruses, which, after infecting cells, produce large concentrations of antigens that eventually trigger an immune response [84]. Due to the high transduction efficiency of adenoviruses, there is great interest in engineering them as vectors for carrying genes/mRNA, although this would depend on the presence of specific receptors for internalization [85]. The genes coding for these viruses are partially or completely replaced by the desired antigen genes, and these modified viruses act as vectors for the delivery of these nucleic acid cargoes. It is noteworthy that positive-strand RNA viruses, such as alphaviruses [86], picornaviruses [87], and flaviviruses [88] have been used to deliver mRNA. The replicative features of positive-strand RNA viruses cause these saRNA vector systems to act as adjuvants, leading to high and transient expression of exogenous proteins [14]. However, the use of viral vectors poses a number of challenges, including genome integration, toxicity, and immunogenicity [89]. For this reason, the use of non-viral vectors based on polymers, lipids, etc. as mRNA delivery systems has advantages [90].

#### 3.3.3. Lipid-Based Carriers

Different synthetic and natural lipids can be used to deliver nucleic acids [91]. The lipids used for the delivery of mRNA vaccines are in the forms of liposomes or lipid nanoparticles (LNPs). Liposomes have long been used as carriers for drugs due to their desirable properties, such as their easy preparation and low toxicity [92]. A variety of liposomes have been designed and studied for the delivery of mRNA vaccines and have shown promise for use in the treatment of diseases such as cancer [93]. The term lipoplexe refers to a nucleic acid and liposome complex. During a self-assembly process, cationic liposomes form complexes with RNA through electrostatic interactions, leading to the formation of lipoplexes [91]. 1,2-di-*O*-octadecenyl-3-trimethylammonium-propane (DOTMA) and 1,2-dioleoyl-3-trimethylammoniumpropane (DOTAP) are examples of cationic lipids that are used to deliver mRNA vaccines [93]. In addition, neutral lipids are used in cationic liposomes to achieve high transfection and low toxicity [94]. Therefore, the activity and functional properties of lipoplexes are related to various factors, including the overall particle charge, lipid content, and the cationic lipid to mRNA ratio [95]. The results of previous studies show that lipoplexes encoding viral antigens or neoantigens elicit memory T cell responses and prevent tumor progression. The two challenges for cationic lipids are their rapid clearance and high toxicity. These disadvantages can be overcome by replacing them with ionizable lipids [96]. At acidic and physiological pHs, ionizable lipids have positive and neutral charges, respectively. This can help them to maintain their transfection efficiency and reduce their toxicity, but these lipids are not widely used in lipoplex formulations and should be used in LNPs [97].

LNPs are one the most broadly used tools for in vivo mRNA delivery [98]. They usually consist of four components: an ionizable or cationic lipid, cholesterol, phospholipids, and lipid-linked polyethylene glycol (PEG). As previously mentioned, ionizable lipids are positively charged under acidic conditions, which leads to the encapsulation of RNA, and at physiological pH, they have a neutral or partial cationic charge. This property is essential, as it allows mRNA to escape from the endosome and be released into the cytoplasm [99]. Cholesterol, as a stabilizing agent, and phospholipid, as a supporting agent for the formation of the lipid bilayer structure, are components of LNPs. Polyethylene glycol increases the LNP circulation time because it prevents the binding of mRNA to proteins in plasma [100]. LNPs have been used to deliver mRNA vaccines that act against viral infections, such as the Zika virus [101], and for cancer immunotherapies, such as that used to treat B16F10 melanoma [102]. The use of LNPs to deliver mRNA encoding antigens that act against B16F10 melanoma tumors has been associated with a reduction in tumor size and an increase in survival following the induced immune response.

#### 3.3.4. Polymer-Based Carriers

Polymer-based carriers have been used less frequently in clinical trials than lipids; however, they have significant potential for use in nucleic acid delivery. Polymers include cationic and anionic structures; however, the use of cationic polymers as nucleic acid carriers is more applicable. Cationic polymers form complexes with anionic mRNA via electrostatic interactions [57]. Polyplex nanoparticles and micelleplex nanoparticles result from such interactions and have differences from and similarities with each other [103]. Polyethylenimine (PEI) is the most widely used cationic polymer and the most commonly used transfection agent for nucleic acids [104,105]. Polyethylenimine has a branched structure with a high cationic charge density [104]. The presence of several amine groups in the polyethylenimine backbone as well as other cationic polymers means that it can bind easily to nucleic acid phosphate groups and form Polyplex nanoparticles, which increases the transfection efficiency and mRNA protection [106]. Cationic polymers establish electrostatic interactions with the negatively charged endosome membrane through these amine groups which contribute to endosomal escape and mRNA release into the cytosol. In addition, the buffering capacity of amine groups on cationic polymers causes a proton-sponge effect that finally induces osmotic swelling and destruction of the endosome membrane [104,107].

In a study of mRNA vaccines, a polyethylenimine-polyplex nanoparticle containing mRNA encoding the influenza virus hemagglutinin and nucleocapsid was used. In this study, mRNA was successfully delivered to dendritic cells, transferred to the cytosol, and translated into proteins, leading to both humoral and cellular immune reactions [108]. However, highly positively charged polyethylene-based formulations have increased toxicity, as they bind to negatively charged serum proteins; therefore, other cationic polymers have been developed, including poly(2-dimethylaminoethyl methacrylate) (PDMAEMA) [109], polyamidoamine (PAMAM) dendrimer [110], biodegradable poly(β-amino ester) (PBAE) [111], poly(amino-co-ester) (PACE) [112], and polysaccharide [113] polymers. Although cationic polymers have more applications than anionic ones, anionic polymers such as poly d,l-lactide-co-glycolide may sometimes be used. However, cationic lipids need to be added to establish a stable complex with negatively charged mRNA [114].

Micelleplexes are another polymer-based nucleic acid delivery system form. They consist of a hydrophobic inner core and a hydrophilic outer shell. In fact, micelleplexes are produced by amphiphilic copolymers consisting of one or more cationic blocks, and due to having a positive charge, they interact with negatively charged nucleic acids and form stable complexes. Micelleplexes have a remarkable ability to co-deliver drugs (such as chemotherapeutic agents) and nucleic acids [115,116]. The first micelleplex-based delivery system for mRNA vaccines was developed using branched PEI2k and stearic acid conjugates (PSA). These PSA/mRNA micelles can be effectively transmitted into cells and escape endosomally. In addition, they are better at inducing dendritic cell maturation and immune profiles than PEI/mRNA complexes, indicating that these nanomicelles have the potential for vaccine delivery [117].

Research related to polymer-based delivery systems is in the early preclinical trial stage. Thus far, these polymer-based carriers have been shown to offer a promising platform for the effective delivery of mRNA.

#### 3.3.5. Hybrid Carriers

Carrier systems for delivering mRNA vaccines may involve a combination of several different substances. These hybrid carriers include lipopolyplexes and cationic nanoemulsions. Lipopolyplexes consist of an inner core, a complex containing a nucleic acid and polycationic component (cationic polymer or cationic peptide), and an outer lipid shell [118,119,120]. These hybrid carriers are actually a combination of a lipoplex and polyplex and offer more advantages than non-hybrid systems [121]. Poly(lactide-co-glycolide) (PLGA), polycaprolactone, and polylactic acid are examples of polymers, and DOTAP, 1,2-dilauroyl-sn-glycero-3-phosphocholine, 1,2-distearoyl-sn-glycero-3-phosphocholine, lecithin, DSPE, and PEG are examples of lipids that are mostly used in the formulation of hybrid nanoparticles [122]. One study showed that the use of histidylated lipopolyplexes of mRNA encoding MART1 in tumor models significantly prevents the growth and progression of B16F10 melanoma tumors and induces a cellular immune response [123]. These results indicate that the use of this type of mRNA formulation as a vaccine is more efficient than using a lipoplex and polyplex alone.

Cationic nanoemulsions (CNE) are another form of hybrid vector with other structural compounds in addition to lipids and polymers. The Novartis Institute developed an MF59-based cationic nanoemulsion method to self-amplify mRNA vaccine delivery [124]. MF59 is a proprietary immunologic adjuvant based on squalene. The cationic nanoemulsion has an oil phase consisting of squalene, DOTAP, and sorbitan trioleate. The addition of DOTAP allows electrostatical binding to mRNA. This oil phase combines with an aqueous phase, which consists of compounds such as Tween 80 [125]. The use of these cationic nanoemulsions as carriers of saRNA vaccines has been associated with an increase in immunogenicity. The advantage of cationic nanoemulsions is the use of oils and surfactants that have already been used successfully in clinical trials and whose safety has been proven [126].

#### 3.3.6. Peptide-Based Carriers

Peptides can be used as non-viral mRNA delivery systems because of their relative stability, low immunogenicity, and low toxicity [127]. Cationic peptides easily interact with negatively charged mRNA due to their positive charge resulting from the amino groups contained in their amino acids [128]. Protamines and cell-penetrating peptides (CPPs) are two important cationic peptides that can be used to deliver mRNA to cells. A desirable feature of protamine is the protection of mRNA from degradation by serum nucleases [129]. However, due to the extremely tight connection of protamine with mRNA, the use of protamine–mRNA complexes alone prevents the translation process and reduces the effectiveness of a vaccine. This can be compensated for by using RNActive technology in which the mRNA–protamine complex acts as a stimulant of the immune system and does not play a role in the expression system [130,131]. In fact, with this platform, the vaccine consists of two compounds: naked mRNA and mRNA complexed with protamine in which naked mRNA is expressed to the desired antigen. The mRNA–protamine complex acts as an adjuvant that induces an adaptive immune response through TLR7-mediated signaling [130,132].

Another study used the fusion of protamines and a class of CCP for mRNA transfection to encode reporter genes into human cells [133]. Cell-penetrating peptides are small peptides, 8 to 30 amino acids in length, that have the ability to penetrate biological membranes and transfer cargo to cellular targets [134]. These peptides are divided into three categories: natural protein-derived peptides, chimeric peptides, and synthetic peptides. Cell-penetrating peptides have been shown to be highly effective for transfection. Some can escape from the endosome through the proton-sponge effect, which leads to osmotic swelling and rupture of the endosome, and some, through interaction with the membrane, can cause its destruction and pore formation [135,136]. Fusogenic lipids, such as dioleoylphosphatidylethanolamine (DOPE), are commonly added to CPPs to enhance endosomal escape.

Anionic peptides can also be used in delivery systems, but because the negative peptide charge and negative mRNA charge repel each other, a cationic copolymer is needed to encapsulate the mRNA. For example, the pHDPA copolymer is used to encapsulate OVA-mRNA, which is then conjugated to an anionic peptide, GALA. Anionic peptides aid in the process of cell uptake via sialic acid on dendritic cells. This vector has been associated with an increase in transfection and the induction of an immune response [137].

#### 3.3.7. Dendritic-Cell-Based mRNA Vaccines

As the most potent antigen-presenting cells in the immune system, dendritic cells (DCs) can internalize, process, and present antigens to CD8^+^ or CD4^+^ T cells on major histocompatibility complexes (MHCs), including MHC class I and MHC class II. Due to the prominent biological features of DCs that are associated with initiation of the adaptive immune response, DCs are no doubt an ideal vaccine target to prevent cancers. Moreover, DCs secrete various kinds of cytokines and chemokines, which are indispensable for T cell proliferation, activation, and recruitment [138,139]. Thus, numerous preclinical and clinical trials have been initiated in previous decades to assess DC-based mRNA vaccines. There are two approaches for the delivery of DC-based mRNA vaccines, loading DCs ex vivo and targeting DCs in vivo, as we described previously [140]. The advantages of ex vivo DC loading possesses are precise antigen stimulation, superior cellular condition control, and high transfection efficiency. However, the major obstacles to the development of such vaccination methods are the labor-intensive procedures and high costs involved. Although in vivo targeting methods are superior in terms of rapid manufacture and low costs, specific and efficient cell-type delivery is still hard to reach. These two delivery approaches have been investigated, and some promising results have been achieved recently.

Myeloid DCs and plasmacytoid DCs represent the two major types of DCs found in the peripheral blood and the most potent antigen-presenting cells in the immune system. Nevertheless, these two kinds of DC constitute only 0.1% of peripheral blood mononuclear cells (PBMCs), and it is difficult and expensive to obtain a sufficient concentration of DCs for use in vaccination against cancers. The most commonly used approach to generate large numbers of DCs for vaccination is the establishment of monocyte-derived DCs (MDDCs). In this process, immature DCs are differentiated from monocytes in the presence of GM-CSF and IL-4 for five days and then stimulated to mature through the addition of maturation stimuli [141]. Although DC can directly uptake antigen-encoding mRNA molecules via endocytosis, various methodologies have been developed to enhance the internalization efficiency ex vivo, including electroporation, lipofection, nucleofection, and sonoporation [142,143,144]. Among these methods, electroporation is utilized for ex vivo transfection the most frequently. Under a high-voltage pulse, cell membrane pores are formed and mRNA molecules can pass through effortlessly, leading to antigen translation in the cytoplasm [142]. In 2012, Aarntzen et al. electroporated MDDCs with mRNA encoding gp100 and tyrosinase and administered them intranodally to forty-five stage III and IV melanoma patients [145]. Robust tumor antigen-specific CD4^+^ and CD8^+^ T-cell responses were evident, and clinical benefits were observed in this trial, favoring the use of mRNA-electroporated dendritic cell vaccines against melanoma. Lipofection is also an attractive approach for ex vivo DC mRNA vaccine generation. Markov and colleagues reported the application of cationic liposomes for the transfection of mRNA in DCs [146]. The injection of DCs pulsed with cationic liposomes resulted in an obvious suppression of metastasis in a model of murine B16 melanoma in vivo. However, as an adoptive cell transfer technique, ex vivo DC-based mRNA vaccines still face challenges, as their production is time and labor intensive.

In vivo DC-targeting mRNA vaccines are administered intranodally. Previous work showed that intranodally delivered mRNA is almost exclusively taken up and translated by lymph-node-resident DCs [147]. Furthermore, intranodal administration achieves the same level of immunogenicity as other administration routes with lower vaccine doses [59,148]. A completed clinical trial has already revealed the possibility of using in vivo mRNA delivery to DCs through intranodal injection of naked neoepitope-encoding mRNA in advanced melanoma patients [79]. To enhance the stimulation efficiency of in vivo DC targeting mRNA delivery, the use of TriMix, which contains three different mRNA encoding immune-stimulatory proteins—CD40 ligand (CD40L), CD70, and constitutively active Toll-like receptor 4 (TLR4)—as an adjuvant combined with tumor antigen-encoding mRNA was investigated [149]. This combination demonstrated superiority in the stimulation of DCs when administered intranodally [147]. Recently, a preclinical evaluation of mRNA trimannosylated lipopolyplexes revealed that in vivo targeting DCs can be realized through intradermal administration [150]. This delivery system is equipped with mannose-containing glycolipids, which specifically target endocytosis receptors presented on the membranes of DCs, highlighting the in vivo targeting DC potency of this mRNA formulation.

## 4. mRNA Vaccines in Combination with Checkpoint Blockade for the Treatment of Melanoma Cancer

The expression of immune-checkpoint molecules is a mechanism that regulates the functioning of the immune system. Such molecules include cytotoxic T-lymphocyte-associated antigen-4 (CTLA-4) and programmed death-1 receptor (PD-1). Both immune checkpoints are expressed on T cells. CTLA-4 interacts with CD80 and CD86 with a higher affinity than the co-stimulant CD28, which leads to the limited activation of T lymphocytes [151]. PD-1 interacts with programmed death ligands (PD-L1 and PD-L2), which are expressed by a wide range of cells (tumor cells, myeloid dendritic cells derived from monocytes, epithelial cells, T and B lymphocytes) and sends a negative signal to T cells, which leads to the depletion of the latter [152]. Thus, these molecules, along with Treg cells, can suppress the anti-tumor T-cell response.

Immune checkpoint inhibitors have already been licensed as a therapeutic in the form of monoclonal antibodies against CTLA-4, PD-1, and PD-L1. These are aimed, in particular, at combating melanoma [153]. Initially, efforts were directed toward the study of CTLA-4. Preclinical trials have shown that blocking CTLA-4 with monoclonal antibodies leads to a significant delay in tumor growth in murine models of melanoma and many other cancers [154], which is associated with increased T cell infiltration [155]. Based on these results, many clinical trials involving the use of monoclonal antibodies for CTLA-4 inhibition have been performed. One of the first drugs approved for the treatment of patients with metastatic melanoma was Ipilimumab, which increased median overall survival (OS) by 10 months [156].

Preclinical trials of PD-1 and PD-L1 inhibitors have shown favorable therapeutic responses in mice with melanoma [155,157]. The blockage of PD-1 has been shown to enhance CD8^+^ T-cell infiltration by increasing the secretion of IFN-γ-inducible chemokines [158]. This led to clinical trials, including a significant study in 2012, which demonstrated positive results in the treatment of advanced melanoma, non-small cell lung cancer, prostate cancer, renal cell, and colorectal cancer [159]. Currently, Pembrolizumab and Nivolumab have been approved and are now used to treat melanoma [153]. It is important to clarify that the functioning of the considered immune checkpoint inhibitors depends on the stage of T-cell activation; therefore, final effects may vary. Specifically, CTLA-4 limits the early stage of T-cell activation; therefore, the inhibition of CTLA-4 leads to an increase in the effector T-cell pool in the lymph nodes but not in the tumor microenvironment [160]. PD-1 exerts its influence on T cells located in the periphery; therefore, T-lymphocyte infiltration increases at the site of the tumor if PD-1 blockers are utilized [160]. Several studies have been based on this difference. For example, a clinical study was conducted in patients with melanoma to compare Ipilimumab (CTLA-4 blocker) and Pembrolizumab (PD-1 blocker) [161]. The best response was given by patients receiving Pembrolizumab. Another study demonstrated that the use of the combination of Ipilimumab and Nivolumab to treat patients with melanoma yields a more significant response than the use of either separately [162].

Although showing partial effectiveness, immune checkpoint inhibitors affect late-stage antitumor T-cell response. To achieve the best effect, cancer immunotherapies might be combined, while other effects on tumor cells should be taken into account. A cocktail of drugs that affect different signaling pathways is more likely to have a therapeutic effect than the use of the same drugs individually. A study by Spranger et al. showed that the blockade of immune checkpoints cannot restore tumor-specific T-cell responses to completely unrecognized, non-infiltrated melanomas; that is, if they are inhibited, the T-cell response will be nonspecific [163]. The network of suppressive myeloid cells may also represent a barrier to immunotherapy development; therefore, it is likely that the inhibition of colony-stimulating factor 1 receptor (CSF1R) in conjunction with blockade of immune checkpoints may demonstrate a synergistic effect [164].

An example of rational immunotherapeutics combination is the simultaneous application of immune checkpoint inhibitors and cancer vaccines. Recently, various combinations of mRNA vaccines with immune checkpoint inhibitors have been actively explored. The recruitment phase of a clinical trial is underway to analyze whether the combination of mRNA-4157 (mRNA cancer vaccine targeting twenty tumor-associated antigens (TAAs)) with Pembrolizumab will improve relapse-free survival compared with the use of Pembrolizumab alone in patients with complete resection of skin melanoma and a high risk of recurrence (NCT03897881). It has been shown that a potent immune response is generated when Ipilimumab is combined with mRNA encoding TriMix (cluster of differentiation 70, ligand of cluster of differentiation 40, and constitutively active toll-like receptor (4) and TAAs (TriMixDC-MEL) in two phase II clinical studies in patients with stage III/IV melanoma (NCT01676779, NCT01302496)) [165,166]. More recently, BioNTech announced a collaboration with Regeneron to begin a phase II clinical trial in patients with anti-PD1-resistant/recurrent inoperable stage III or IV cutaneous melanoma to monitor the effects of treatment with mRNA (BNT111) encoding four TAAs (NY-ESO-1, MAGE-A3, tyrosinase, and TPTE) in combination with Celiplimab (PD-1 blocker) [167]. Another clinical trial that is currently in the recruiting stage is aiming to compare the use of BMS-986016 (monoclonal antibody against lymphocyte activation gene (3) alone and in combination with Nivolumab (PD-1-blocker) (NCT01968109)). There are now several clinical trials involving mRNA encoding a neoantigen combined with PD-1 inhibitors that are in the recruiting stage (NCT03289962, NCT03815058, NCT03897881). Sahin et al. presented data from a preliminary interim analysis of their study in which patients with inoperable melanoma who were already taking were given a vaccine containing RNA-LPX encoding four TAAs (FixVac) either alone or in combination with PD1 blockade [168]. They observed that although FixVac is active as a separate agent, it also acts synergistically with anti-PD1 therapy in patients who do not respond to checkpoint inhibitor monotherapy.

Another option is the combination of checkpoint blockers with mRNA encoding cytokines to modulate the cytokine environment in the tumor microenvironment. In situ delivery of mRNA encoding a fusokine called Fβ2, which consists of IFN-β fused to the ectodomain of the transforming growth factor-β II receptor, has been performed. It was shown that such constructions can delay tumor growth, and this can be further enhanced by blocking the interaction of PD-1 with PD-L1 [60]. A trial of a combination therapy consisting of mRNA encoding OX40L and Durvalumab is currently in the recruitment stage (NCT03323398). Additionally, the combination of a mixture of IL-23/IL-36γ/OX40L triplet mRNAs with checkpoint blockade has been shown to be more effective in models that are otherwise resistant to the systemic inhibition of immune checkpoints [169].

## 5. Clinical Trials of In Vitro Transcription mRNA Vaccines in Melanoma

Although in vitro transcription (IVT) mRNA antitumor vaccines represent a minority of melanoma immunotherapeutics in clinical trials in comparison with DC-based mRNA vaccines, the promising results and progression of IVT mRNA vaccines collected from preclinical studies indicates their great potential for use as immunotherapies for treating melanoma. In 1990, the first report regarding the successful expression of IVT mRNA transfected in mouse skeletal muscle cells revealed the possibility of converting IVT mRNA into vaccines against infectious diseases or cancers [170]. However, limited advancement in the development of mRNA cancer vaccines has been achieved since then mainly due to concerns about mRNA instability, insufficient translation potency in vivo, and high innate immunogenicity. Owing to the rapid development of molecular biotechnology, the use of IVT mRNA for cancer treatment as a vaccine has become more feasible through modulation of the mRNA structure, purification of mRNA by novel methods, and the formulation of mRNA within delivery vehicles [14]. As pioneers of the pharmaceutical industry, BioNTech, Moderna, and GenenTech have all announced clinical updates on IVT mRNA vaccines against melanoma, as summarized in Table 1. Their representative mRNA-based immunotherapies have been broadly evaluated in clinical trials, and some encouraging results have been obtained, although some are still in the recruiting stage. Another appealing alternative, saRNA, which originates from single-strand mRNA viruses, has also gained significant attention due to its self-amplification property and persistent antigen expression capability [56]. Currently, although saRNA can be synthesized after IVT without viral particle packages, reducing safety concerns regarding the viral components, no saRNA vaccines are being used in clinical trials of melanoma due to the debate over the clinical benefits versus potential immunological damage to patients that such vaccines may cause. Therefore, in this section, we only discuss mRNA vaccines delivered by non-viral vectors, which have been extensively explored recently.

The selection of antigens is a key issue in the establishment and development of cancer vaccines. Various features of antigens, like their immunogenicity and avidity, impact the efficiency of cancer vaccines [171]. The choice of antigen is critical during the design of cancer vaccines to better train and bolster the immune systems of hosts to fight against cancer and eventually eliminate malignant cells. Tumor-associated antigens (TAAs), which have low levels of expression in normal tissues while showing elevated expression in tumor cells, have been well-studied and used in the development of cancer vaccines [172]. Nonetheless, due to their “self-protein” characteristic, TAAs are not completely ideal targets for cancer vaccines. Another emerging target, neoantigens, which originate from non-synonymous mutations in tumor cells and are absent from normal cells, have been applied in cancer vaccine manufacturing and have shown unique advantages in clinical trials, especially for melanoma patients [173,174]. Currently, multiple mRNA-based cancer vaccines, either encoding a cocktail of TAAs or personalized neoantigens, have been assessed in clinical trials of melanoma (Table 1). Furthermore, the versatility of mRNA has paved a path beyond its use as a source of tumor antigens. mRNA encoding immunostimulants, which can induce the maturation and activation of antigen-presenting cells (APCs), promoting T cell-mediated immune responses and modifying the suppressive tumor microenvironment, have been applied as novel immunotherapies to combat cancer in combination with other cancer vaccines or immunotherapeutic agents (e.g., immune checkpoint inhibitors) [15]. This mRNA-encoding immunostimulant strategy has evolved the landscape of mRNA-based cancer vaccines, contributing to the eradication of tumor cells using a comprehensive method.

The early-stage clinical trials of IVT mRNA cancer vaccines against melanoma targeted melanoma-associated antigens (MAAs), which are preferentially expressed in melanoma cells. In 2004, Weide et al. launched a clinical trial in which protamine-stabilized mRNAs encoding Melan-A, Tyrosinase, gp100, Mage-A1, Mage-A3, and Survivin were used to treat melanoma patients (NCT00204607) [175]. After receiving the vaccine intradermally, enhancement of vaccine-specific T-cell immune response was observed in two out of four immunologically evaluable patients. One out of seven stage IV patients with measurable disease showed a complete response during a 36-month observation period. In 2015, BioNTech initiated a multicenter, open-label, dose-escalation phase I trial (Lipo-MERIT, NCT02410733) to assess the efficiency and safety of FixVac (BNT111), which is composed of RNA-LPX encoding NY-ESO-1, MAGE-A3, TPTE, and tyrosinase, for the treatment of advanced melanoma patients [168]. Patients expressing at least one of these MAAs underwent eight vaccination cycles. This well-known nanoparticulate liposomal RNA vaccine displayed its antitumor effects by bolstering the immune response against at least one MAA in 39 out of 50 patients. In the FixVac monotherapy arm (n = 25), three patients achieved a partial response, and seven attained a stable disease state. Seventeen patients received the FixVac plus anti-PD1 antibody in the combination group, and six patients developed a partial response in this arm. Results collected from this trial revealed that the FixVac encoding four types of non-mutant MAAs has clinical benefits for melanoma patients when combined with ICBs, especially for those with a lower tumor mutation burden (TMB).

IVT mRNA vaccines encoding MAAs have already shown their feasibility for use in the treatment of melanoma. However, central tolerance is still the major challenge that threatens the efficiency of mRNA vaccines targeting MAAs. Furthermore, non-mutant MAA mRNA vaccines could theoretically decrease in potency when applied to treat melanoma, which is the TMB tumor type with the greatest magnitude [176]. In this new era of using antigens for the cancer vaccine development, neoantigens have been shown to have advantages in terms of inducing a highly specific, robust anti-tumor immune response with an individual approach. This is in contrast to MAAs, especially for treating high-TMB melanoma. Sahin et al. reported the first-in-human application of an RNA-based polyneoepitope vaccine to treat melanoma (NCT02035956) [79]. Non-synonymous mutations expressed by thirteen patients with melanoma were identified by exome and RNA sequencing, and ten selected mutations per patient were constructed into two synthetic RNAs. Each patient received at least eight doses of the neoepitope mRNA vaccine intranodally. One-third of patients with pre-existing weak responses against neoepitopes showed augmented responses, while two-thirds showed de novo responses. Eight patients without radiologically detectable lesions at the start of the administration period generated vigorous immune responses and experienced the absence of recurrence for a 12–23 month postvaccination follow-up period, while the other five relapsed shortly after inclusion. Interestingly, this trial revealed a stronger neoepitope-specific than TAA-specific response in patients who received both vaccinations, indicating that a lack of central tolerance might be the underlying mechanism.

With the rapid development of nanotechnology, LNP was designed as a powerful delivery vehicle for mRNA vaccines with an effective internalization capability, endosomal escape, and cell/organ-selective targeting. One well-known product named mRNA-4157 was launched by Moderna. This is a personalized mRNA vaccine encapsulated in LNP that is used to treat patients with solid tumors including, but not limited to, melanoma (NCT03897881) [177]. The formulation was found to be well-tolerated and no adverse events were reported in this trial. Twelve out of the thirteen patients in the monotherapy arm retained a disease-free status during an 8 month follow-up period, while in the combination group (n = 20), there was one complete response, two partial responses, and five patients with stable disease for at least five vaccination cycles. Furthermore, BioNTech and GenenTech have also initiated multiple phase I and II trials to assess the efficacy and safety of personalized LNP-encapsulated mRNA vaccines encoding neoantigens (RO7198457, RO7198458) in combination with Atezolizumab or Pembrolizumab (NCT03289962, NCT03815058).

Unlike antigen-targeting vaccines, immunostimulant-encoding vaccines fulfill their function by priming APCs and T cell-mediated immunity and modifying the dysfunctional tumor microenvironment [178,179]. Thus, the evaluation of mRNA encoding immunostimulants as monotherapies or for use in combination therapies with other immunological agents has also been investigated in multiple clinical trials. One pioneer product invented by eTheRNA known as the “TriMix” mRNA adjuvant vaccine, which encodes CD70 to activate CD8^+^ T cells, CD40L to stimulate CD4^+^ T cells, and constitutively active TLR4 to promote antigen presentation in DCs [180], has been proven to be well tolerated and immunogenic in clinical trials against melanoma [181,182,183]. However, TriMix is mainly utilized in ex vivo DC transfection to induce the maturation and activation of DCs as one major step in adoptive DC transfer therapy. Few clinical trials concerning direct TriMix injection have been conducted. One trial aiming to assess the immunogenicity and safety of the ECI006 vaccine (a combination of TriMix and MAA-encoding mRNA) via intranodal administration was initiated in 2017 (NCT03394937) [184]. In this study, no serious adverse events occurred in the postvaccination period, indicating the good tolerability and safety of ECI006. Furthermore, a vaccine-induced immune response was demonstrated in four out of ten and three out of nine patients treated with low and high doses, respectively. More promising clinical results are expected in this ongoing trial. There are also other adjuvant vaccine formulations, like mRNA-2752, a product developed by Moderna, which is composed of OX40L, IL-23, and IL-36. This product has been shown to boost the anticancer response by inducing the activation of T cells and modulating innate and adaptive immunity (NCT03739931). Although the efficacy and tolerability of other adjuvant vaccines against melanoma have not been tested in clinical trials yet, the guaranteed results achieved from other types of cancer highlight its potential effects on melanoma.

## 6. DC mRNA Vaccines in Melanoma

Having an essential role in initiating the specific antitumor immune response, DCs can be utilized to deliver mRNA-encoding tumor antigens in cancer immunotherapies based on their biological features. The first demonstration of adoptive mRNA-pulsed DC transfer was conducted by Boczkowski and colleagues in 1996 [185]. They found that DCs pulsed with ovalbumin (OVA)-encoding mRNA were more effective than OVA peptide-pulsed DCs for priming OVA-specific CTL responses in vitro. Furthermore, mice vaccinated with DCs pulsed with OVA-encoding mRNA were protected from OVA-expressing tumor cells. In their study, a dramatic reduction in lung metastases was observed in the poorly immunogenic, highly metastatic B16/F10.9 tumor model after vaccination with DCs pulsed with tumor-derived mRNA, revealing that DCs pulsed with mRNA represent an attractive platform for cancer treatment and have the potential to be translated into clinical practice. In addition to the ex vivo DC loading approach, in vivo DC targeting is an alternative for the design of DC-based mRNA vaccines. However, this vaccine platform is usually realized by intranodal administration, and the specific targeting of DCs is hard to guarantee due to the diverse environment in lymph nodes. Thus, in this section, we only discuss the use of ex vivo DC loading mRNA vaccines for the treatment of melanoma. Thus far, clinical trials concerning DC-based mRNA vaccines have been initiated for patients with various types of cancer, including colorectal cancer [186], glioblastoma [187], prostate cancer [188], and acute myeloid leukemia [189].

Notably, DC-based mRNA vaccines have recently been widely applied to treat melanoma patients, and promising results have been achieved from these clinical trials (Table 2). Gaudernack and colleagues extracted autologous total mRNA from tumor biopsies of melanoma patients and then electroporated it into DCs [190,191]. In vivo, vaccine-specific immune responses elicited by DCs transfected with autologous tumor-derived mRNA have been shown to be evident in melanoma patients. Although a broad spectrum of T cell responses has been demonstrated after vaccination with tumor-derived mRNA transfected DCs, tumor-derived mRNA generation requires a large number of tumor biopsies, which means melanoma patients are usually in the late stage of disease, and limited effects can be achieved by vaccine administration. Furthermore, tumor-derived mRNA also encodes common host antigens; thus, the cytotoxicity against cancer prompted by mRNA encoding tumor antigens is weakened by central tolerance. To achieve a more specific immune response toward melanoma and avoid potential adverse events, TAA-encoding mRNA, including MAGE-A3, MAGE-C2, tyrosinase, and gp100, is mostly chosen for electroporation into DCs. Interestingly, unlike tyrosinase and gp100, which are widely represented in melanoma but also expressed in normal tissues, MAGE-A3 and MAGE-C2, as cancer-germline antigens, are exclusively expressed in germ cells as well as in various types of cancers including, but not limited to, melanoma [192,193]. The existence of the blood–testis barrier prevents the recognition of cancer-germline antigens by the immune system. Once these antigens are move beyond the testis, a robust immune response against them is aroused. This biological feature endows cancer-germline antigens great potential for use in vaccine design [171,194]. In Wilgenhof’s trial, a specific MAGE-A3 and MAGE-C2 immune response was demonstrated in the postvaccination period in 7 and 10 out of 21 patients, respectively. This was superior to the responses of tyrosinase and gp100, revealing the potency of cancer-germline antigens and their potential for use in mRNA vaccines against melanoma [195]. In addition, as a pharmacological and immunological agent that modifies the effect of the vaccine, immunological adjuvants function by stimulating and amplifying immune responses to targeted antigens but do not confer immunity themselves. The utilized adjuvant for DC-based mRNA vaccines against melanoma that has been used most commonly in clinical trials is TriMix. This mRNA-based adjuvant contains three naked mRNA molecules, which encode the activation stimulator CD40 ligand (CD40L), which activates CD4^+^ T cells; the costimulatory molecule CD70, which activates CD8^+^ T cells; and constitutively active TLR4, which promotes DC antigen presentation [196]. The use of the TriMix adjuvant in combination with antigen-encoding mRNA has been evaluated in multiple DC-based mRNA vaccine clinical trials and has been proven to be well-tolerated and immunogenic [181,182,195,197,198].

All clinical trials indicated in Table 2 were performed using electroporation as the mRNA transfection method for DCs. Electroporation is now a mature technique, and its transfection potency has been demonstrated in various preclinical and clinical trials. Nevertheless, other emerging transfection platforms, such as lipofection, nucleofection, and sonoporation, are considered alternatives for electroporation, and further clinical trial results are required to assess the transfection efficiency and biosafety of these methods [143,144]. For DC-based mRNA vaccines, intravenous and intradermal injections are regular administration routes that have been widely applied in clinical trials. Several studies have shown that only a small proportion of injected DCs reach the regional lymph nodes after intradermal administration [199,200]. Therefore, intranodal injection seems to be advantageous for DC-based vaccine administration compared with intradermal injection.

Interestingly, Gaudernack et al. reported no difference between intranodal and intradermal injection in relation to eliciting immune responses in the postvaccination period [190]. This might be because (1) having a small percentage of successfully migrating DCs is sufficient for T cell activation in the lymph nodes, (2) successfully migrating DCs already receive further maturation signals during migration, and (3) damage to the lymph node structure occurs during intranodal administration.

DC-based mRNA vaccines have shown promising antitumor effects against melanoma in clinical trials. In 2011, Wilgenhof et al. reported that when TriMixDC/IFN-α-2b combination therapy was applied to advanced melanoma patients, one partial response was observed and 5 patients achieved a stable disease status out of 17 patients. The median OS and progression-free survival (PFS) were 15.1 months and 3.1 months, respectively [195]. In 2013, Wilgenhof et al. reported on a phase Ib study in which intravenous TriMixDC-MEL pulsed with MAAs was used to treat advanced melanoma patients. In this trial, two partial responses were achieved and 2 patients attained a stable disease status out of 15 patients. The median PFS and OS were 5 and 14 months, respectively [181]. In 2020, a randomized controlled phase II clinical trial was conducted by Jansen and colleagues. In that study, the one-year disease-free survival rate was 71% in the TriMixDC-MEL arm and 35% in the control arm. The time to non-salvageable recurrence or death was longer in the TriMixDC-MEL arm than in the control arm [166]. These encouraging results from clinical trials provide a solid foundation for the development of DC-based mRNA vaccines in clinical practice. However, more firm clinical outcomes, especially from randomized two-arm studies, are urgently needed. Moreover, to circumvent the limitations of monoimmunotherapies in cancer treatment, a rational DC-based mRNA vaccine combination approach requires further investigation. Such an approach could impact the tumor and modulate the dysfunctional tumor microenvironment in a comprehensive manner.

## 7. Therapeutic Considerations, Challenges, and Future Directions

Although mRNA vaccines have many advantages for the treatment of cancer, they are still in the early stages of research. Currently, it is necessary to take all aspects related to the safety of a product into account, including the above-mentioned problems associated with the immunogenicity, stabilization, and efficacy of mRNA vaccines. The development of an autoimmune response against the background of the immune-stimulating function of mRNA vaccines is a significant clinical problem. The identification of highly immunogenic tumor-specific antigens is challenging, since tumor antigens may differ greatly among individuals. Finally, since antitumor vaccines are therapeutic rather than prophylactic in most cases, and since cancer treatment requires long-term multiple administrations of high-dose medicaments, the safety requirements for the creation and use of mRNA vaccines must be significantly tightened. Nonetheless, mRNA vaccines have a promising future and great potential to become one of the main strategies for cancer treatment. One of the important advantages of mRNA cancer vaccines is their fast and scalable production, allowing the product to be produced in a short time. This advantage is especially important due to the frequent rapid disease progression in cancer patients.

The most important area of research in the cancer vaccine therapy field is the advancement of the identification of individual cancer neoantigens derived from mutations or other abnormal sequences that are technically challenging to identify. Therefore, further studies to improve sequencing and bioinformatic approaches to analyze antigens and their epitopes as well as predict their binding to MHC molecules are of considerable importance. Further improvement to mRNA cancer vaccines could be obtained through combinations with complementary immunotherapeutic approaches. Based on successful results obtained from the combination of cancer vaccines with immune checkpoint inhibitors, combination therapies should be considered as prominent approaches for cancer treatments, and further experiments should be conducted in this area.

Overall, the number of ongoing studies in the field of mRNA cancer vaccines is growing significantly (Table 1 and Table 2). The experience gained through the development of mRNA vaccines to prevent COVID-19 will undoubtedly play a role in the development of mRNA cancer vaccines as well, especially in regard to production protocols, storage, and distribution. Low costs, manufacturing benefits, and the ability to tailor personalized preparations will pave the way for the production of mRNA vaccines.

## Figures and Tables

**Table 1:** Clinical trials of IVT mRNA-based vaccines for the treatment of melanoma.

Encoding Content	Trial ID	Start Date	Phase	Enrollment Status	Brand	Target Antigens	Formulation	Route	Combination	Study Results	Sponsor
MAAs	NCT 00204607	Jun 2004	I/II	Completed	NA	Melan-A, MAGE-A1, MAGE-A3, Survivin, GP100, Tyrosinase	Protamine-protected mRNA	i.d.	GM-CSF	An increase of vaccine-specific T cells was observed in two of four immunologically evaluable patients. One of seven patients with measurable diseases showed a CR.	University Hospital Tuebingen
	NCT 00204516	Apr 2007	I/II	Completed	NA	Melan-A, MAGE-A1, MAGE-A3, Survivin, GP100, Tyrosinase	Naked mRNA	i.d.	GM-CSF		University Hospital Tuebingen
	NCT 01684241	Jun 2012	I	Completed		NY-ESO-1, tyrosinase	Naked mRNA	i.n.	None		BioNTech
	NCT 02410733	Mar 2015	I	Active, not recruiting	FixVac (BNT111)	NY-ESO-1, MAGE-A3, TPTE, tyrosinase	RNA-LPX	i.v.	Anti-PD1	More than 75% showed immune responses against at least one MAA in 50 patients. In the FixVac monotherapy group (n = 25), three patients experienced a PR and seven had SD, while in the FixVac/anti-PD1 combination group, 6 out of 17 patients developed a PR.	BioNTech
Neoantigens	NCT 02035956	Oct 2013	I	Completed	IVACMUTANOME	Neopeptides	Naked mRNA	i.n.	mRNA encoding NY-ESO-1, tyrosinase	One-third of pre-existing weak responses against neo-epitopes were augmented while two-thirds were de novo responses. 13 patients in total. Eight patients without measurable lesions at the start of the trial developed robust immune responses against neo-epitopes and achieved recurrence-free for the whole follow-up period.	BioNTech
	NCT 03289962	Dec 2017	I	Recruiting	RO7198457	Neopeptides	LNP	i.n.	Atezolizumab		BioNTech GenenTech
	NCT 03815058	Jan 2019	II	Recruiting	RO7198458	Neopeptides	LNP	i.v.	Pembrolizumab		BioNTech GenenTech
	NCT 03480152	Nov 2019	I/II	Terminated	mRNA-4650	Neopeptides	LNP	i.m.	None		Moderna
	NCT 03897881	Jul 2019	II	Recruiting	mRNA-4157	Neopeptides	LNP	i.v.	Pembrolizumab	No ≥ grade III AEs occurred. Of the 13 patients on monotherapy, 12 patients remain disease-free. In the combination group (n = 20), one CR, two PR, and five SD were observed for at least five administration cycles.	Moderna Merck
Immunostimulants	NCT 03394937	Jun 2017	I	Recruiting	ECI-006	CD70, CD40L, caTLR4	Naked mRNA	i.n.	mRNA encoding tyrosinase, gp100, MAGE-A3, MAGE-C2, PRAME	No AEs Grade 3 or higher were reported. Vaccine-induced immune responses were detected in 4/10 and 3/9 patients treated with the low and high dose, respectively.	eTheRNA immunotherapies

Abbreviation: MAAs: Melanoma-associated antigens; RNA-LPX: liposomal RNA; LNP: Lipid nanoparticle; AEs: Adverse events; CR: Complete response; PR: Partial response; SD: Stable disease; i.v.: Intravenous; i.d.: Intradermal; i.n.: Intranodal; i.m.: intramuscular; NA: Not available.

**Table 2:** Completed clinical trials of DC-based mRNA vaccines against melanoma.

Year	Trial ID	Phase	Antigen	Formulation	Route	Combination	Grade ≥3 Adverse Events	Study Results	Refs
2006	NA	I/II	Autologous tumor-mRNA	Electroporation	i.d. and i.n.	None	None	A vaccine-specific immune response was demonstrated in 9/19 patients evaluated by T-cell assays and in 8/18 patients evaluated by DTH reaction. The response rates do not suggest an advantage in applying i.n. vaccination compared with i.d. vaccination.	[190]
2007	NA	I/II	Autologous tumor-mRNA	Electroporation	i.d. and i.n.	None	None	The immunological data indicated sustained T cell responses and suggested an enhancing effect of booster vaccinations.	[191]
2011	NA	NA	MAGE-A3, MAGE-C2, tyrosinase, gp100	Electroporation with TriMix-mRNA	i.d.	IFN-α-2b	None	Vaccinal antigen-specific DIL were found in 0/6 patients tested at vaccine initiation and in 12/21 (57.1%) assessed after the fourth vaccine. During TriMixDC/IFN-a-2b combination therapy, one PR and five SD were observed in 17 patients with evaluable disease at baseline.	[195]
2012	NA	NA	MAGE-A3, MAGE-C2, tyrosinase, gp100	Electroporation with TriMix-mRNA	i.v. and i.d.	None	NA	Ex vivo-generated mRNA-modified DCs can induce effector CD8^+^ and CD4^+^ T cells from the naive T-cell repertoire of melanoma patients.	[197]
2013	NCT 01066390	Ib	MAGE-A3, MAGE-C2, tyrosinase, gp100	Electroporation with TriMix-mRNA	i.v.	None	None	In a total of 15 patients, two patients achieved a CR and two patients a PR. All objective responders achieved a PFS. Antigen-specific SKILs were documented in 6 of 12 patients, and antigen-specific CD8^+^ T-cells were detected in the blood of four of five patients.	[181]
2015	NA	NA	MAGE-A1, MAGE-A3, MAGE-C2, MelanA/MART-1, tyrosinase, gp100	Electroporation with TriMix-mRNA	i.d.	IFN-α-2b	None	The median relapse-free survival is 22 months (95 % CI 12–32 months), the 2-year and 4-year survival rates are 93% and 70%, respectively.	[182]
2016	NCT 01302496	II	MAGE-A3, MAGE-C2, tyrosinase, gp100	Electroporation with TriMix-mRNA	i.v. and i.d.	Ipilimumab	17	The 6-month disease control rate was 51% (95% CI, 36% to 67%), and the overall tumor response rate was 38%, seven CR and one PR are ongoing after a median follow-up time of 36 months	[183]
2020	NCT 01676779	II	MAGEA3, MAGE-C2, tyrosinase, gp100	Electroporation with TriMix-mRNA	i.v. and i.d.	None	None	71% of patients in the study arm were free of disease compared with 35% in the control arm after one year. The median time to non-salvageable recurrence was superior in the study arm.	[198]
2020	NCT 02285413	II	tyrosinase, gp100	Electroporation	i.v. and i.d.	Cisplatin	1	Antigen-specific CD8^+^ T cells were found in 44% versus 67%, and functional T cell responses in 28% versus 19% in patients receiving DC vaccination with and without cisplatin, respectively. A significantly better OS is observed in stage III patients treated with combination therapy compared with DC monotherapy.	[166]

Abbreviation: CR: Complete response; PR: Partial response; SD: Stable disease; PFS: Progression-free survival; i.v.: Intravenous; i.d.: Intradermal; i.n.: Intranodal; DTH: Delayed-type hypersensitivity; DIL: DTH-infiltrating lymphocytes; MAGE: Melanoma-associated antigen; NA: No available.

## Data Availability

Not applicable.

## References

[1] American Cancer Society Cancer Facts & Statistics. http://cancerstatisticscenter.cancer.org/.

[2] Ulmer A., Dietz K., Hodak I., Polzer B., Scheitler S., Yildiz M., Czyz Z., Lehnert P., Fehm T., Hafner C. (2014). Quantitative Measurement of Melanoma Spread in Sentinel Lymph Nodes and Survival. PLoS Med..

[3] Payandeh Z., Yarahmadi M., Nariman-Saleh-Fam Z., Tarhriz V., Islami M., Aghdam A.M., Eyvazi S. (2019). Immune therapy of melanoma: Overview of therapeutic vaccines. J. Cell. Physiol..

[4] Reiman J.M., Kmieciak M., Manjili M.H., Knutson K.L. (2007). Tumor immunoediting and immunosculpting pathways to cancer progression. Semin. Cancer Biol..

[5] Hazarika M., Chuk M.K., Theoret M.R., Mushti S., He K., Weis S.L., Putman A.H., Helms W.S., Cao X., Li H. (2017). U.S. FDA Approval Summary: Nivolumab for Treatment of Unresectable or Metastatic Melanoma Following Progression on Ipilimumab. Clin. Cancer Res..

[6] Barone A., Hazarika M., Theoret M.R., Mishra-Kalyani P., Chen H., He K., Sridhara R., Subramaniam S., Pfuma E., Wang Y. (2017). FDA Approval Summary: Pembrolizumab for the Treatment of Patients with Unresectable or Metastatic Melanoma. Clin. Cancer Res..

[7] Postow M.A., Chesney J., Pavlick A.C., Robert C., Grossmann K., McDermott D., Linette G.P., Meyer N., Giguere J.K., Agarwala S.S. (2015). Nivolumab and Ipilimumab versus Ipilimumab in Untreated Melanoma. N. Engl. J. Med..

[8] Pardoll D.M. (2012). The blockade of immune checkpoints in cancer immunotherapy. Nat. Rev. Cancer.

[9] Frey A.B. (2015). Suppression of T cell responses in the tumor microenvironment. Vaccine.

[10] Thomas S., Prendergast G.C. (2016). Cancer Vaccines: A Brief Overview. Methods Mol. Biol..

[11] Alexandrov L., Initiative A.P.C.G., Nik-Zainal S., Wedge D., Aparicio S.A.J.R., Behjati S., Biankin A., Bignell G.R., Bolli N., Borg A. (2013). Signatures of mutational processes in human cancer. Nature.

[12] Weber J.S., D’Angelo S.P., Minor D., Hodi F.S., Gutzmer R., Neyns B., Hoeller C., I Khushalani N., Miller W.H., Lao C.D. (2015). Nivolumab versus chemotherapy in patients with advanced melanoma who progressed after anti-CTLA-4 treatment (CheckMate 037): A randomised, controlled, open-label, phase 3 trial. Lancet Oncol..

[13] Robert C., Long G., Brady B., Dutriaux C., Maio M., Mortier L., Hassel J.C., Rutkowski P., McNeil C., Kalinka E. (2015). Nivolumab in Previously Untreated Melanoma withoutBRAFMutation. N. Engl. J. Med..

[14] Pardi N., Hogan M.J., Porter F.W., Weissman D. (2018). mRNA vaccines—A new era in vaccinology. Nat. Rev. Drug Discov..

[15] Van Hoecke L., Verbeke R., Dewitte H., Lentacker I., Vermaelen K., Breckpot K., Van Lint S. (2021). mRNA in cancer immunotherapy: Beyond a source of antigen. Mol. Cancer.

[16] Hodi F.S. (2006). Well-Defined Melanoma Antigens as Progression Markers for Melanoma: Insights into Differential Expression and Host Response Based on Stage. Clin. Cancer Res..

[17] Pitcovski J., Shahar E., Aizenshtein E., Gorodetsky R. (2017). Melanoma antigens and related immunological markers. Crit. Rev. Oncol..

[18] Ordóñez N.G. (2014). Value of melanocytic-associated immunohistochemical markers in the diagnosis of malignant melanoma: A review and update. Hum. Pathol..

[19] Rosenberg S.A. (2001). Progress in human tumour immunology and immunotherapy. Nature.

[20] Barrow C., Browning J., MacGregor D., Davis I.D., Sturrock S., Jungbluth A.A., Cebon J. (2006). Tumor Antigen Expression in Melanoma Varies According to Antigen and Stage. Clin. Cancer Res..

[21] Fogal S., Carotti M., Giaretta L., Lanciai F., Nogara L., Bubacco L., Bergantino E. (2015). Human Tyrosinase Produced in Insect Cells: A Landmark for the Screening of New Drugs Addressing its Activity. Mol. Biotechnol..

[22] Hearing V.J. (2005). Biogenesis of pigment granules: A sensitive way to regulate melanocyte function. J. Dermatol. Sci..

[23] Kobayashi H., Lu J., Celis E. (2001). Identification of helper T-cell epitopes that encompass or lie proximal to cytotoxic T-cell epitopes in the gp100 melanoma tumor antigen. Cancer Res..

[24] Gjerstorff M.F., Kock K., Nielsen O., Ditzel H.J. (2007). MAGE-A1, GAGE and NY-ESO-1 cancer/testis antigen expression during human gonadal development. Hum. Reprod..

[25] Cronwright G., Le Blanc K., Götherström C., Darcy P., Ehnman M., Brodin B. (2005). Cancer/Testis Antigen Expression in Human Mesenchymal Stem Cells: Down-regulation of SSX Impairs Cell Migration and Matrix Metalloproteinase 2 Expression. Cancer Res..

[26] Tandler N., Mosch B., Pietzsch J. (2012). Protein and non-protein biomarkers in melanoma: A critical update. Amino Acids.

[27] Robbins P.F., Kassim S.H., Tran T.L.N., Crystal J.S., Morgan R.A., Feldman S.A., Yang J.C., Dudley M.E., Wunderlich J.R., Sherry R.M. (2015). A Pilot Trial Using Lymphocytes Genetically Engineered with an NY-ESO-1–Reactive T-cell Receptor: Long-term Follow-up and Correlates with Response. Clin. Cancer Res..

[28] Johnson L.A., Morgan R.A., Dudley M.E., Cassard L., Yang J.C., Hughes M.S., Kammula U.S., Royal R.E., Sherry R.M., Wunderlich J.R. (2009). Gene therapy with human and mouse T-cell receptors mediates cancer regression and targets normal tissues expressing cognate antigen. Blood.

[29] Walker E.B., Haley D., Miller W., Floyd K., Wisner K.P., Sanjuan N., Maecker H.T., Romero P., Hu H.-M., Alvord W.G. (2004). gp100209–2M Peptide Immunization of Human Lymphocyte Antigen-A2+ Stage I-III Melanoma Patients Induces Significant Increase in Antigen-Specific Effector and Long-Term Memory CD8+ T Cells. Clin. Cancer Res..

[30] Gubin M.M., Artyomov M.N., Mardis E.R., Schreiber R.D. (2015). Tumor neoantigens: Building a framework for personalized cancer immunotherapy. J. Clin. Investig..

[31] Cantwell-Dorris E.R., O’Leary J., Sheils O. (2011). BRAFV600E: Implications for Carcinogenesis and Molecular Therapy. Mol. Cancer Ther..

[32] Boespflug A., Caramel J., Dalle S., Thomas L. (2017). Treatment of NRAS-mutated advanced or metastatic melanoma: Rationale, current trials and evidence to date. Ther. Adv. Med. Oncol..

[33] Fecek R.J., Storkus W.J. (2016). Combination strategies to enhance the potency of monocyte-derived dendritic cell-based cancer vaccines. Immunotherapy.

[34] Ott P.A., Shuqiang L., Keskin D.B., Shukla S.A., Sun J., Bozym D.J., Zhang W., Luoma A., Giobbie-Hurder A., Peter L. (2017). An immunogenic personal neoantigen vaccine for patients with melanoma. Nat. Cell Biol..

[35] Rubinsteyn A., Kodysh J., Hodes I., Mondet S., Aksoy B.A., Finnigan J.P., Bhardwaj N., Hammerbacher J. (2017). Computational Pipeline for the PGV-001 Neoantigen Vaccine Trial. Front. Immunol..

[36] Hayward N., Wilmott J., Waddell N., Johansson P.A., Field M., Nones K., Patch A.-M., Kakavand H., Alexandrov L.B., Burke H. (2017). Whole-genome landscapes of major melanoma subtypes. Nature.

[37] Kramps T., Elbers K. (2017). Introduction to RNA Vaccines. RNA Vaccines.

[38] Desfarges S., Ciuffi A. (2012). Viral Integration and Consequences on Host Gene Expression. Viruses: Essential Agents of Life.

[39] Iavarone C., O’hagan D., Yu D., Delahaye N., Ulmer J. (2017). Mechanism of action of mRNA-based vaccines. Expert Rev. Vacc..

[40] Lundstrom K. (2020). Self-amplifying RNA viruses as RNA vaccines. Int. J. Mol. Sci..

[41] Deering R.P., Kommareddy S., Ulmer J.B., Brito L.A., Geall A.J. (2014). Nucleic acid vaccines: Prospects for non-viral delivery of mRNA vaccines. Expert Opin. Drug Deliv..

[42] Beissert T., Perkovic M., Vogel A., Erbar S., Walzer K.C., Hempel T., Brill S., Haefner E., Becker R., Türeci Ö. (2019). A Trans-amplifying RNA Vaccine Strategy for Induction of Potent Protective Immunity. Mol. Ther..

[43] Hornung V., Barchet W., Schlee M., Hartmann G. (2008). RNA recognition via TLR7 and TLR8. Toll-Like Receptors (TLRs) and Innate Immunity. Handb. Exp. Pharmacol..

[44] Nelson J., Sorensen E.W., Mintri S., Rabideau A.E., Zheng W., Besin G., Khatwani N., Su S.V., Miracco E.J., Issa W.J. (2020). Impact of mRNA chemistry and manufacturing process on innate immune activation. Sci. Adv..

[45] Alexopoulou L., Holt A.C., Medzhitov R., Flavell R.A. (2001). Recognition of double-stranded RNA and activation of NF-κB by Toll-like receptor 3. Nature.

[46] Weng Y., Li C., Yang T., Hu B., Zhang M., Guo S., Xiao H., Liang X.-J., Huang Y. (2020). The challenge and prospect of mRNA therapeutics landscape. Biotechnol. Adv..

[47] Thess A., Grund S., Mui B.L., Hope M.J., Baumhof P., Fotin-Mleczek M., Schlake T. (2015). Sequence-engineered mRNA without Chemical Nucleoside Modifications Enables an Effective Protein Therapy in Large Animals. Mol. Ther..

[48] Mitchell P., Tollervey D. (2000). mRNA stability in eukaryotes. Curr. Opin. Genet. Dev..

[49] Wilson T.M.A., Treisman R. (1988). Removal of poly(A) and consequent degradation of c-fos mRNA facilitated by 3′ AU-rich sequences. Nature.

[50] Wilusz C.J., Wormington M., Peltz S.W. (2001). The cap-to-tail guide to mRNA turnover. Nat. Rev. Mol. Cell Biol..

[51] Kudla G., Lipinski L., Caffin F., Helwak A., Zylicz M. (2006). High Guanine and Cytosine Content Increases mRNA Levels in Mammalian Cells. PLoS Biol..

[52] Gallie D.R. (1991). The cap and poly(A) tail function synergistically to regulate mRNA translational efficiency. Genes Dev..

[53] Martin S., Paoletti E., Moss B. (1975). Purification of mRNA guanylyltransferase and mRNA (guanine-7-) methyltransferase from vaccinia virions. J. Biol. Chem..

[54] Stepinski J., Waddell C., Stolarski R., Darzynkiewicz E., Rhoads R.E. (2001). Synthesis and properties of mRNAs containing the novel “anti-reverse” cap analogs 7-methyl (3’-O-methyl) GpppG and 7-methyl (3’-deoxy) GpppG. Rna.

[55] Weissman D., Karikó K. (2015). mRNA: Fulfilling the Promise of Gene Therapy. Mol. Ther..

[56] Miao L., Zhang Y., Huang L. (2021). mRNA vaccine for cancer immunotherapy. Mol. Cancer.

[57] Kowalski P., Rudra A., Miao L., Anderson D.G. (2019). Delivering the Messenger: Advances in Technologies for Therapeutic mRNA Delivery. Mol. Ther..

[58] Pardi N., Tuyishime S., Muramatsu H., Kariko K., Mui B.L., Tam Y.K., Madden T.D., Hope M.J., Weissman D. (2015). Expression kinetics of nucleoside-modified mRNA delivered in lipid nanoparticles to mice by various routes. J. Control. Release.

[59] Senti G., Kündig T.M. (2015). Intralymphatic immunotherapy. World Allergy Organ. J..

[60] Van Der Jeught K., Joe P.T., Bialkowski L., Heirman C., Daszkiewicz L., Liechtenstein T., Escors D., Thielemans K., Breckpot K. (2014). Intratumoral administration of mRNA encoding a fusokine consisting of IFN-β and the ectodomain of the TGF-β receptor II potentiates antitumor immunity. Oncotarget.

[61] Holtkamp S., Kreiter S., Selmi A., Simon P., Koslowski M., Huber C., Türeci O., Sahin U. (2006). Modification of antigen-encoding RNA increases stability, translational efficacy, and T-cell stimulatory capacity of dendritic cells. Blood.

[62] Ziemniak M., Strenkowska M., Kowalska J., Jemielity J. (2013). Potential therapeutic applications of RNA cap analogs. Futur. Med. Chem..

[63] Malone R.W., Felgner P.L., Verma I.M. (1989). Cationic liposome-mediated RNA transfection. Proc. Natl. Acad. Sci. USA.

[64] Conry R.M., LoBuglio A.F., Wright M., Sumerel L., Pike M.J., Johanning F., Benjamin R., Lu D., Curiel D.T. (1995). Characterization of a messenger RNA polynucleotide vaccine vector. Cancer Res..

[65] Kuhn A.N., Beißert T., Simon P., Vallazza B., Buck J., Davies B.P., Tureci O., Sahin U. (2012). mRNA as a versatile tool for exogenous protein expression. Curr. Gene Ther..

[66] Ross J., Sullivan T.D. (1985). Half-lives of beta and gamma globin messenger RNAs and of protein synthetic capacity in cultured human reticulocytes. Blood.

[67] Chen C.-Y.A., Shyu A.-B. (1995). AU-rich elements: Characterization and importance in mRNA degradation. Trends Biochem. Sci..

[68] Cannarozzi G., Schraudolph N.N., Faty M., von Rohr P., Friberg M.T., Roth A.C., Gonnet P., Gonnet G., Barral Y. (2010). A Role for Codon Order in Translation Dynamics. Cell.

[69] Gustafsson C., Govindarajan S., Minshull J. (2004). Codon bias and heterologous protein expression. Trends Biotechnol..

[70] Kimchi-Sarfaty C., Oh J., Kim I.-W., Sauna Z.E., Calcagno A.M., Ambudkar S.V., Gottesman M.M. (2007). A “Silent” Polymorphism in the MDR1 Gene Changes Substrate Specificity. Science.

[71] Zhong F., Cao W., Chan E., Tay P.N., Cahya F.F., Zhang H., Lu J. (2005). Deviation from major codons in the Toll-like receptor genes is associated with low Toll-like receptor expression. Immunology.

[72] Karikó K., Muramatsu H., Keller J.M., Weissman D. (2012). Increased Erythropoiesis in Mice Injected with Submicrogram Quantities of Pseudouridine-containing mRNA Encoding Erythropoietin. Mol. Ther..

[73] Van Gulck E.R.A., Ponsaerts P., Heyndrickx L., Vereecken K., Moerman F., De Roo A., Colebunders R., Bosch G.V.D., Van Bockstaele D.R., Van Tendeloo V.F.I. (2006). Efficient stimulation of HIV-1-specific T cells using dendritic cells electroporated with mRNA encoding autologous HIV-1 Gag and Env proteins. Blood.

[74] Diken M., Kreiter S., Selmi A., Britten C.M., Huber C., Tureci O., Sahin U. (2011). Selective uptake of naked vaccine RNA by dendritic cells is driven by macropinocytosis and abrogated upon DC maturation. Gene Ther..

[75] Tan L., Sun X. (2018). Recent advances in mRNA vaccine delivery. Nano Res..

[76] Ringer S. (1882). Regarding the Action of Hydrate of Soda, Hydrate of Ammonia, and Hydrate of Potash on the Ventricle of the Frog’s Heart. J. Physiol..

[77] Lee J.A. (1981). Sydney Ringer (1834–1910) and Alexis Hartmann (1898–1964). Anaesthesia.

[78] Probst J., Weide B., Scheel B., Pichler B.J., Hoerr I., Rammensee H.-G., Pascolo S. (2007). Spontaneous cellular uptake of exogenous messenger RNA in vivo is nucleic acid-specific, saturable and ion dependent. Gene Ther..

[79] Sahin U., Derhovanessian E., Miller M., Kloke B.-P., Simon P., Löwer M., Bukur V., Tadmor A.D., Luxemburger U., Schrörs B. (2017). Personalized RNA mutanome vaccines mobilize poly-specific therapeutic immunity against cancer. Nature.

[80] Wang Y., Zhang Z., Luo J., Han X., Wei Y., Wei X. (2021). mRNA vaccine: A potential therapeutic strategy. Mol. Cancer.

[81] Lorenz C., Fotin-Mleczek M., Roth G., Becker C., Dam T.C., Verdurmen W.P.R., Brock R., Probst J., Schlake T. (2011). Protein expression from exogenous mRNA: Uptake by receptor-mediated endocytosis and trafficking via the lysosomal pathway. RNA Biol..

[82] Selmi A., Vascotto F., Kautz-Neu K., Türeci Ö., Sahin U., Von Stebut E., Diken M., Kreiter S. (2016). Uptake of synthetic naked RNA by skin-resident dendritic cells via macropinocytosis allows antigen expression and induction of T-cell responses in mice. Cancer Immunol. Immunother..

[83] Stewart M.P., Langer R., Jensen K.F. (2018). Intracellular Delivery by Membrane Disruption: Mechanisms, Strategies, and Concepts. Chem. Rev..

[84] Wadhwa A., Aljabbari A., Lokras A., Foged C., Thakur A. (2020). Opportunities and challenges in the delivery of mRNA-based vaccines. Pharmaceutics.

[85] Chou J.Y., Mansfield B.C. (2011). Recombinant AAV-directed gene therapy for type I glycogen storage diseases. Expert Opin. Biol. Ther..

[86] Ehrengruber M.U., Schlesinger S., Lundstrom K. (2011). Alphaviruses: Semliki Forest virus and Sindbis virus vectors for gene transfer into neurons. Curr. Protoc. Neurosci..

[87] Rozovics J.M., Chase A.J., Cathcart A.L., Chou W., Gershon P.D., Palusa S., Wilusz J., Semler B.L. (2012). Picornavirus Modification of a Host mRNA Decay Protein. mBio.

[88] Schott J.W., Morgan M., Galla M., Schambach A. (2016). Viral and Synthetic RNA Vector Technologies and Applications. Mol. Ther..

[89] Tezel A., Dokka S., Kelly S., Hardee G.E., Mitragotri S. (2004). Topical Delivery of Anti-sense Oligonucleotides Using Low-Frequency Sonophoresis. Pharm. Res..

[90] Ramamoorth M., Narvekar A. (2015). Non viral vectors in gene therapy—An overview. J. Clin. Diagn. Res. JCDR.

[91] Midoux P., Pichon C. (2014). Lipid-based mRNA vaccine delivery systems. Expert Rev. Vaccines.

[92] Torchilin V.P. (2005). Recent advances with liposomes as pharmaceutical carriers. Nat. Rev. Drug Discov..

[93] Kranz L., Diken M., Haas H., Kreiter S., Loquai C., Reuter K.C., Meng M., Fritz D., Vascotto F., Hefesha H. (2016). Systemic RNA delivery to dendritic cells exploits antiviral defence for cancer immunotherapy. Nature.

[94] Lv H., Zhang S., Wang B., Cui S., Yan J. (2006). Toxicity of cationic lipids and cationic polymers in gene delivery. J. Control. Release.

[95] Grabbe S., Haas H., Diken M., Kranz L.M., Langguth P., Sahin U. (2016). Translating nanoparticulate-personalized cancer vaccines into clinical applications: Case study with RNA-lipoplexes for the treatment of melanoma. Nanomedicine.

[96] Hajj K.A., Whitehead K.A. (2017). Tools for translation: Non-viral materials for therapeutic mRNA delivery. Nat. Rev. Mater..

[97] Reichmuth A.M., Oberli M.A., Jaklenec A., Langer R., Blankschtein D. (2016). mRNA vaccine delivery using lipid nanoparticles. Ther. Deliv..

[98] Lokugamage M., Gan Z., Zurla C., Levin J., Islam F., Kalathoor S., Sato M., Sago C.D., Santangelo P.J., Dahlman J.E. (2019). Mild Innate Immune Activation Overrides Efficient Nanoparticle-Mediated RNA Delivery. Adv. Mater..

[99] Ramishetti S., Hazan-Halevy I., Palakuri R., Chatterjee S., Naidu Gonna S., Dammes N., Freilich I., Kolik Shmuel L., Danino D., Peer D. (2020). A combinatorial library of lipid nanoparticles for RNA delivery to leukocytes. Adv. Mater..

[100] Samaridou E., Heyes J., Lutwyche P. (2020). Lipid nanoparticles for nucleic acid delivery: Current perspectives. Adv. Drug Deliv. Rev..

[101] Pardi N., Hogan M., Pelc R., Muramatsu H., Andersen H., DeMaso C.R., Dowd K.A., Sutherland L.L., Scearce R.M., Parks R. (2017). Zika virus protection by a single low-dose nucleoside-modified mRNA vaccination. Nature.

[102] Oberli M.A., Reichmuth A.M., Dorkin J.R., Mitchell M., Fenton O.S., Jaklenec A., Anderson D.G., Langer R., Blankschtein D. (2016). Lipid Nanoparticle Assisted mRNA Delivery for Potent Cancer Immunotherapy. Nano Lett..

[103] Gary D.J., Lee H., Sharma R., Lee J.-S., Kim Y., Cui Z.Y., Jia D., Bowman V.D., Chipman P.R., Wan L. (2011). Influence of Nano-Carrier Architecture on In Vitro siRNA Delivery Performance and In Vivo Biodistribution: Polyplexes vs Micelleplexes. ACS Nano.

[104] Boussif O., Lezoualc’H F., Zanta M.A., Djavaheri-Mergny M., Scherman D., Demeneix B., Behr J.P. (1995). A versatile vector for gene and oligonucleotide transfer into cells in culture and In Vivo: Polyethylenimine. Proc. Natl. Acad. Sci. USA.

[105] Lungwitz U., Breunig M., Blunk T., Göpferich A. (2005). Polyethylenimine-based non-viral gene delivery systems. Eur. J. Pharm. Biopharm..

[106] Howard K.A., Rahbek U.L., Liu X., Damgaard C., Glud S.Z., Andersen M., Hovgaard M.B., Schmitz A., Nyengaard J.R., Besenbacher F. (2006). RNA Interference in Vitro and in Vivo Using a Novel Chitosan/siRNA Nanoparticle System. Mol. Ther..

[107] Akinc A., Thomas M., Klibanov A.M., Langer R. (2005). Exploring polyethylenimine-mediated DNA transfection and the proton sponge hypothesis. J. Gene Med. A Cross-Discip. J. Res. Sci. Gene Transf. Its Clin. Appl..

[108] Démoulins T., Milona P., Englezou P.C., Ebensen T., Schulze K., Suter R., Pichon C., Midoux P., Guzmán C.A., Ruggli N. (2016). Polyethylenimine-based polyplex delivery of self-replicating RNA vaccines. Nanomed. Nanotechnol. Biol. Med..

[109] Üzgün S., Nica G., Pfeifer C., Bosinco M., Michaelis K., Lutz J.-F., Schneider M., Rosenecker J., Rudolph C. (2011). PEGylation Improves Nanoparticle Formation and Transfection Efficiency of Messenger RNA. Pharm. Res..

[110] Vaidyanathan S., Orr B.G., Holl M.M.B. (2016). Role of Cell Membrane–Vector Interactions in Successful Gene Delivery. Acc. Chem. Res..

[111] Yin H., Kanasty R.L., Eltoukhy A.A., Vegas A.J., Dorkin J.R., Anderson D.G. (2014). Non-viral vectors for gene-based therapy. Nat. Rev. Genet..

[112] Jiang Y., Gaudin A., Zhang J., Agarwal T., Song E., Kauffman A.C., Tietjen G.T., Wang Y., Jiang Z., Cheng C.J. (2018). A “top-down” approach to actuate poly(amine-co-ester) terpolymers for potent and safe mRNA delivery. Biomaterials.

[113] Stefan J., Kus K., Wisniewska A., Lorkowska-Zawicka B., Kaminski K., Szczubialka K., Nowakowska M., Korbut R. (2019). The antiatherogenic effect of new biocompatible cationically modified polysaccharides: Chitosan and pullulan—The comparison study. J. Physiol. Pharmacol. Off. J. Pol. Physiol. Soc..

[114] Yang X.-Z., Dou S., Sun T.-M., Mao C., Wang H., Wang J. (2011). Systemic delivery of siRNA with cationic lipid assisted PEG-PLA nanoparticles for cancer therapy. J. Control. Release.

[115] Almeida M., Magalhães M., Veiga F., Figueiras A. (2018). Poloxamers, poloxamines and polymeric micelles: Definition, structure and therapeutic applications in cancer. J. Polym. Res..

[116] Jhaveri A., Torchilin V.P. (2014). Multifunctional polymeric micelles for delivery of drugs and siRNA. Front. Pharmacol..

[117] Zhao M., Li M., Zhang Z., Gong T., Sun X. (2015). Induction of HIV-1 gag specific immune responses by cationic micelles mediated delivery of gag mRNA. Drug Deliv..

[118] Perche F., Benvegnu T., Berchel M., Lebegue L., Pichon C., Jaffrès P.-A., Midoux P. (2011). Enhancement of dendritic cells transfection in vivo and of vaccination against B16F10 melanoma with mannosylated histidylated lipopolyplexes loaded with tumor antigen messenger RNA. Nanomed. Nanotechnol. Biol. Med..

[119] Persano S., Guevara M.L., Li Z., Mai J., Ferrari M., Pompa P.P., Shen H. (2017). Lipopolyplex potentiates anti-tumor immunity of mRNA-based vaccination. Biomaterials.

[120] Rezaee M., Oskuee R.K., Nassirli H., Malaekeh-Nikouei B. (2016). Progress in the development of lipopolyplexes as efficient non-viral gene delivery systems. J. Control. Release.

[121] Guan S., Rosenecker J. (2017). Nanotechnologies in delivery of mRNA therapeutics using nonviral vector-based delivery systems. Gene Ther..

[122] Colombo S., Cun D., Remaut K., Bunker M., Zhang J., Martin-Bertelsen B., Yaghmur A., Braeckmans K., Nielsen H.M., Foged C. (2015). Mechanistic profiling of the siRNA delivery dynamics of lipid–polymer hybrid nanoparticles. J. Control. Release.

[123] Mockey M., Bourseau E., Chandrashekhar V., Chaudhuri A., Lafosse S., Le Cam E., Quesniaux V.F.J., Ryffel B., Pichon C., Midoux P. (2007). mRNA-based cancer vaccine: Prevention of B16 melanoma progression and metastasis by systemic injection of MART1 mRNA histidylated lipopolyplexes. Cancer Gene Ther..

[124] Brito L.A., Chan M., Shaw C.A., Hekele A., Carsillo T., Schaefer M., Archer J., Seubert A., Otten G.R., Beard C.W. (2014). A Cationic Nanoemulsion for the Delivery of Next-generation RNA Vaccines. Mol. Ther..

[125] Ott G., Barchfeld G.L., Chernoff D., Radhakrishnan R., van Hoogevest P., Van Nest G. (1995). MF59 Design and Evaluation of a Safe and Potent Adjuvant for Human Vaccines. Vaccine Des..

[126] Lovelyn C., Attama A.A. (2011). Current state of nanoemulsions in drug delivery. J. Biomater. Nanobiotechnol..

[127] Hoyer J., Neundorf I. (2012). Peptide Vectors for the Nonviral Delivery of Nucleic Acids. Acc. Chem. Res..

[128] Qiu Y., Man R.C., Liao Q., Kung K.L., Chow M.Y., Lam J.K. (2019). Effective mRNA pulmonary delivery by dry powder formulation of PEGylated synthetic KL4 peptide. J. Control. Release.

[129] Hoerr I., Obst R., Rammensee H.G., Jung G. (2000). In Vivo application of RNA leads to induction of specific cytotoxic T lymphocytes and antibodies. Eur. J. Immunol..

[130] Kallen K.-J., Heidenreich R., Schnee M., Petsch B., Schlake T., Thess A., Baumhof P., Scheel B., Koch S.D., Fotin-Mleczek M. (2013). A novel, disruptive vaccination technology: Self-adjuvanted RNActive^®^ vaccines. Hum. Vaccines Immunother..

[131] Schlake T., Thess A., Fotin-Mleczek M., Kallen K.-J. (2012). Developing mRNA-vaccine technologies. RNA Biol..

[132] Kallen K.-J., Theß A. (2014). A development that may evolve into a revolution in medicine: mRNA as the basis for novel, nucleotide-based vaccines and drugs. Ther. Adv. Vaccines.

[133] Bell G.D., Yang Y., Leung E., Krissansen G.W. (2018). mRNA transfection by a Xentry-protamine cell-penetrating peptide is enhanced by TLR antagonist E6446. PLoS ONE.

[134] Udhayakumar V.K., De Beuckelaer A., McCaffrey J., McCrudden C.M., Kirschman J.L., Vanover D., Van Hoecke L., Roose K., Deswarte K., De Geest B.G. (2017). Arginine-Rich Peptide-Based mRNA Nanocomplexes Efficiently Instigate Cytotoxic T Cell Immunity Dependent on the Amphipathic Organization of the Peptide. Adv. Health Mater..

[135] Baru M., Nahum O., Jaaro H., Sha’Anani J., Nur I. (1998). Lysosome-disrupting Peptide Increases the Efficiency of In-VivoGene Transfer by Liposome-encapsulated DNA. J. Drug Target..

[136] Wyman T.B., Nicol F., Zelphati O., Scaria P.V., Plank C., Szoka F.C. (1997). Design, Synthesis, and Characterization of a Cationic Peptide That Binds to Nucleic Acids and Permeabilizes Bilayers. Biochemistry.

[137] Lou B., De Koker S., Lau C.Y., Hennink W.E., Mastrobattista E. (2018). mRNA Polyplexes with Post-Conjugated GALA Peptides Efficiently Target, Transfect, and Activate Antigen Presenting Cells. Bioconjugate Chem..

[138] Guermonprez P., Valladeau J., Zitvogel L., Théry C., Amigorena S. (2002). Antigenpresentation Andt Cellstimulation Bydendriticcells. Annu. Rev. Immunol..

[139] Del Prete A., Sozio F., Barbazza I., Salvi V., Tiberio L., Laffranchi M., Gismondi A., Bosisio D., Schioppa T., Sozzani S. (2020). Functional Role of Dendritic Cell Subsets in Cancer Progression and Clinical Implications. Int. J. Mol. Sci..

[140] Baldin A.V., Savvateeva L.V., Bazhin A.V., Zamyatnin J.A.A. (2020). Dendritic Cells in Anticancer Vaccination: Rationale for Ex Vivo Loading or In Vivo Targeting. Cancers.

[141] Sabado R.L., Meseck M., Bhardwaj N., Thomas S. (2016). Dendritic cell vaccines. Vaccine Design: Methods and Protocols: Volume 1: Vaccines for Human Diseases.

[142] Ahmed R., Sayegh N., Graciotti M., Kandalaft L.E. (2020). Electroporation as a method of choice to generate genetically modified dendritic cell cancer vaccines. Curr. Opin. Biotechnol..

[143] Melhem N.M., Gleason S.M., Liu X.D., Boyes S.B. (2008). High-Level Antigen Expression and Sustained Antigen Presentation in Dendritic Cells Nucleofected with Wild-Type Viral mRNA but Not DNA. Clin. Vaccine Immunol..

[144] De Temmerman M.-L., Dewitte H., Vandenbroucke R., Lucas B., Libert C., Demeester J., De Smedt S., Lentacker I., Rejman J. (2011). mRNA-Lipoplex loaded microbubble contrast agents for ultrasound-assisted transfection of dendritic cells. Biomaterials.

[145] Aarntzen E.H.J.G., Schreibelt G., Bol K.F., Lesterhuis W.J., Croockewit A.J., De Wilt J.H.W., Van Rossum M.M., Blokx W., Jacobs H., Boer T.D.-D. (2012). Vaccination with mRNA-Electroporated Dendritic Cells Induces Robust Tumor Antigen-Specific CD4+ and CD8+ T Cells Responses in Stage III and IV Melanoma Patients. Clin. Cancer Res..

[146] Markov O.O., Mironova N., Maslov M., Petukhov I.A., Morozova N.G., Vlassov V., Zenkova M.A. (2012). Novel cationic liposomes provide highly efficient delivery of DNA and RNA into dendritic cell progenitors and their immature offsets. J. Control. Release.

[147] Van Lint S., Goyvaerts C., Maenhout S., Goethals L., Disy A., Benteyn D., Pen J., Bonehill A., Heirman C., Breckpot K. (2012). Preclinical Evaluation of TriMix and Antigen mRNA-Based Antitumor Therapy. Cancer Res..

[148] Johansen P., Häffner A.C., Koch F., Zepter K., Erdmann I., Maloy K., Simard J.J., Storni T., Senti G., Bot A. (2005). Direct intralymphatic injection of peptide vaccines enhances immunogenicity. Eur. J. Immunol..

[149] Bonehill A., Van Nuffel A., Corthals J., Tuyaerts S., Heirman C., François V., Colau D., Van Der Bruggen P., Neyns B., Thielemans K. (2009). Single-Step Antigen Loading and Activation of Dendritic Cells by mRNA Electroporation for the Purpose of Therapeutic Vaccination in Melanoma Patients. Clin. Cancer Res..

[150] Le Moignic A., Malard V., Benvegnu T., Lemiègre L., Berchel M., Jaffrès P.-A., Baillou C., Delost M., Macedo R., Rochefort J. (2018). Preclinical evaluation of mRNA trimannosylated lipopolyplexes as therapeutic cancer vaccines targeting dendritic cells. J. Control. Release.

[151] Van Der Merwe P.A., Bodian D.L., Daenke S., Linsley P., Davis S.J. (1997). CD80 (B7-1) Binds Both CD28 and CTLA-4 with a Low Affinity and Very Fast Kinetics. J. Exp. Med..

[152] Dyck L., Mills K.H. (2017). Immune checkpoints and their inhibition in cancer and infectious diseases. Eur. J. Immunol..

[153] Vaddepally R.K., Kharel P., Pandey R., Garje R., Chandra A.B. (2020). Review of Indications of FDA-Approved Immune Checkpoint Inhibitors per NCCN Guidelines with the Level of Evidence. Cancers.

[154] Van Elsas A., Hurwitz A.A., Allison J.P. (1999). Combination Immunotherapy of B16 Melanoma Using Anti–Cytotoxic T Lymphocyte–Associated Antigen 4 (Ctla-4) and Granulocyte/Macrophage Colony-Stimulating Factor (Gm-Csf)-Producing Vaccines Induces Rejection of Subcutaneous and Metastatic Tumors Accompanied by Autoimmune Depigmentation. J. Exp. Med..

[155] Duraiswamy J., Kaluza K.M., Freeman G.J., Coukos G. (2013). Dual Blockade of PD-1 and CTLA-4 Combined with Tumor Vaccine Effectively Restores T-Cell Rejection Function in Tumors. Cancer Res..

[156] Hodi F.S., O’Day S.J., McDermott D.F., Weber R.W., Sosman J.A., Haanen J.B., Gonzalez R., Robert C., Schadendorf D., Hassel J.C. (2010). Improved Survival with Ipilimumab in Patients with Metastatic Melanoma. N. Engl. J. Med..

[157] Duraiswamy J., Freeman G.J., Coukos G. (2013). Therapeutic PD-1 Pathway Blockade Augments with Other Modalities of Immunotherapy T-Cell Function to Prevent Immune Decline in Ovarian Cancer. Cancer Res..

[158] Peng W., Liu C., Xu C., Lou Y., Chen J., Yang Y., Yagita H., Overwijk W.W., Lizée G., Radvanyi L. (2012). PD-1 Blockade Enhances T-cell Migration to Tumors by Elevating IFN-γ Inducible Chemokines. Cancer Res..

[159] Topalian S.L., Hodi F.S., Brahmer J.R., Gettinger S.N., Smith D.C., McDermott D.F., Powderly J.D., Carvajal R.D., Sosman J.A., Atkins M.B. (2012). Safety, activity, and immune correlates of anti–PD-1 antibody in cancer. N. Engl. J. Med..

[160] Buchbinder E.I., Desai A. (2016). CTLA-4 and PD-1 pathways: Similarities, differences, and implications of their inhibition. Am. J. Clin. Oncol..

[161] Robert C., Ribas A., Schachter J., Arance A., Grob J.-J., Mortier L., Daud A., Carlino M.S., McNeil C.M., Lotem M. (2019). Pembrolizumab versus ipilimumab in advanced melanoma (KEYNOTE-006): Post-hoc 5-year results from an open-label, multicentre, randomised, controlled, phase 3 study. Lancet Oncol..

[162] Larkin J., Chiarion-Sileni V., Gonzalez R., Grob J.-J., Rutkowski P., Lao C.D., Cowey C.L., Schadendorf D., Wagstaff J., Dummer R. (2019). Five-Year Survival with Combined Nivolumab and Ipilimumab in Advanced Melanoma. N. Engl. J. Med..

[163] Spranger S., Bao R., Gajewski T.F. (2015). Melanoma-intrinsic β-catenin signalling prevents anti-tumour immunity. Nature.

[164] Ai M., Curran M.A. (2015). Immune checkpoint combinations from mouse to man. Cancer Immunol. Immunother..

[165] De Keersmaecker B., Claerhout S., Carrasco J., Bar I., Corthals J., Wilgenhof S., Neyns B., Thielemans K. (2020). TriMix and tumor antigen mRNA electroporated dendritic cell vaccination plus ipilimumab: Link between T-cell activation and clinical responses in advanced melanoma. J. Immunother. Cancer.

[166] Jansen Y., Kruse V., Corthals J., Schats K., Van Dam P.-J., Seremet T., Heirman C., Brochez L., Kockx M., Thielemans K. (2020). A randomized controlled phase II clinical trial on mRNA electroporated autologous monocyte-derived dendritic cells (TriMixDC-MEL) as adjuvant treatment for stage III/IV melanoma patients who are disease-free following the resection of macrometastases. Cancer Immunol. Immunother..

[167] Shi Y. (2020). Clinical Translation of Nanomedicine and Biomaterials for Cancer Immunotherapy: Progress and Perspectives. Adv. Ther..

[168] Sahin U., Oehm P., Derhovanessian E., Jabulowsky R.A., Vormehr M., Gold M., Maurus D., Schwarck-Kokarakis D., Kuhn A.N., Omokoko T. (2020). An RNA vaccine drives immunity in checkpoint-inhibitor-treated melanoma. Nature.

[169] Hewitt S.L., Bai A., Bailey D., Ichikawa K., Zielinski J., Karp R., Apte A., Arnold K., Zacharek S.J., Iliou M.S. (2019). Durable anticancer immunity from intratumoral administration of IL-23, IL-36γ, and OX40L mRNAs. Sci. Transl. Med..

[170] Wolff J.A., Malone R.W., Williams P., Chong W., Acsadi G., Jani A., Felgner P.L. (1990). Direct Gene Transfer into Mouse Muscle In Vivo. Science.

[171] Zhao Y., Baldin A., Isayev O., Werner J., Zamyatnin A., Bazhin A. (2021). Cancer Vaccines: Antigen Selection Strategy. Vaccines.

[172] Liu C.-C., Yang H., Zhang R., Zhao J.-J., Hao D.-J. (2016). Tumour-associated antigens and their anti-cancer applications. Eur. J. Cancer Care.

[173] Yarchoan M., Johnson B.A., Lutz E.R., Laheru D.A., Jaffee E.M. (2017). Targeting neoantigens to augment antitumour immunity. Nat. Rev. Cancer.

[174] Li L., Goedegebuure S., Gillanders W. (2017). Preclinical and clinical development of neoantigen vaccines. Ann. Oncol..

[175] Weide B., Pascolo S., Scheel B., Derhovanessian E., Pflugfelder A., Eigentler T., Pawelec G., Hoerr I., Rammensee H.-G., Garbe C. (2009). Direct Injection of Protamine-protected mRNA: Results of a Phase 1/2 Vaccination Trial in Metastatic Melanoma Patients. J. Immunother..

[176] McNamara M.G., Jacobs T., Lamarca A., Hubner R.A., Valle J.W., Amir E. (2020). Impact of high tumor mutational burden in solid tumors and challenges for biomarker application. Cancer Treat. Rev..

[177] Burris H.A., Patel M.R., Cho D.C., Clarke J.M., Gutierrez M., Zaks T.Z., Frederick J., Hopson K., Mody K., Binanti-Berube A. (2019). A phase I multicenter study to assess the safety, tolerability, and immunogenicity of mRNA-4157 alone in patients with resected solid tumors and in combination with pembrolizumab in patients with unresectable solid tumors. J. Clin. Oncol..

[178] Hadden J.W. (1993). Immunostimulants. Trends Pharmacol. Sci..

[179] Galluzzi L., Humeau J., Buqué A., Zitvogel L., Kroemer G. (2020). Immunostimulation with chemotherapy in the era of immune checkpoint inhibitors. Nat. Rev. Clin. Oncol..

[180] Bonehill A., Tuyaerts S., Van Nuffel A., Heirman C., Bos T.J., Fostier K., Neyns B., Thielemans K. (2008). Enhancing the T-cell Stimulatory Capacity of Human Dendritic Cells by Co-electroporation With CD40L, CD70 and Constitutively Active TLR4 Encoding mRNA. Mol. Ther..

[181] Wilgenhof S., Van Nuffel A., Benteyn D., Corthals J., Aerts C., Heirman C., Van Riet I., Bonehill A., Thielemans K., Neyns B. (2013). A phase IB study on intravenous synthetic mRNA electroporated dendritic cell immunotherapy in pretreated advanced melanoma patients. Ann. Oncol..

[182] Wilgenhof S., Corthals J., Van Nuffel A., Benteyn D., Heirman C., Bonehill A., Thielemans K., Neyns B. (2014). Long-term clinical outcome of melanoma patients treated with messenger RNA-electroporated dendritic cell therapy following complete resection of metastases. Cancer Immunol. Immunother..

[183] Wilgenhof S., Corthals J., Heirman C., Van Baren N., Lucas S., Kvistborg P., Thielemans K., Neyns B. (2016). Phase II Study of Autologous Monocyte-Derived mRNA Electroporated Dendritic Cells (TriMixDC-MEL) Plus Ipilimumab in Patients with Pretreated Advanced Melanoma. J. Clin. Oncol..

[184] Arance Fernandez A.M., Baurain J.-F., Vulsteke C., Rutten A., Soria A., Carrasco J., Neyns B., De Keersmaecker B., Van Assche T., Lindmark B. (2019). A phase I study (E011-MEL) of a TriMix-based mRNA immunotherapy (ECI-006) in resected melanoma patients: Analysis of safety and immunogenicity. J. Clin. Oncol..

[185] Boczkowski D., Nair S.K., Snyder D., Gilboa E. (1996). Dendritic cells pulsed with RNA are potent antigen-presenting Cells In Vitro and In Vivo. J. Exp. Med..

[186] Lesterhuis W.J., De Vries I.J.M., Schreibelt G., Schuurhuis D.H., Aarntzen E.H., De Boer A., Scharenborg N.M., Van De Rakt M., Hesselink E.J., Figdor C. (2010). Immunogenicity of dendritic cells pulsed with CEA peptide or transfected with CEA mRNA for vaccination of colorectal cancer patients. Anticancer. Res..

[187] Vik-Mo E.O., Nyakas M., Mikkelsen B.V., Moe M.C., Due-Tønnessen P., Suso E.M.I., Sæbøe-Larssen S., Sandberg C., Brinchmann J.E., Helseth E. (2013). Therapeutic vaccination against autologous cancer stem cells with mRNA-transfected dendritic cells in patients with glioblastoma. Cancer Immunol. Immunother..

[188] Kongsted P., Borch T.H., Ellebaek E., Iversen T.Z., Andersen R., Met Ö., Hansen M., Lindberg H., Sengeløv L., Svane I.M. (2017). Dendritic cell vaccination in combination with docetaxel for patients with metastatic castration-resistant prostate cancer: A randomized phase II study. Cytotherapy.

[189] Van Tendeloo V.F., Van de Velde A., Van Driessche A., Cools N., Anguille S., Ladell K., Gostick E., Vermeulen K., Pieters K., Nijs G. (2010). Induction of complete and molecular remissions in acute myeloid leukemia by Wilms’ tumor 1 antigen-targeted dendritic cell vaccination. Proc. Natl. Acad. Sci. USA.

[190] Kyte J.A., Mu L., Aamdal S., Kvalheim G., Dueland S., Hauser M.A., Gullestad H.P., Ryder T., Lislerud K., Hammerstad H. (2006). Phase I/II trial of melanoma therapy with dendritic cells transfected with autologous tumor-mRNA. Cancer Gene Ther..

[191] Kyte J.A., Kvalheim G., Lislerud K., Straten P.T., Dueland S., Aamdal S., Gaudernack G. (2006). T cell responses in melanoma patients after vaccination with tumor-mRNA transfected dendritic cells. Cancer Immunol. Immunother..

[192] Yao J., Caballero O.L., Yung W.A., Weinstein J.N., Riggins G.J., Strausberg R.L., Zhao Q. (2014). Tumor Subtype-Specific Cancer–Testis Antigens as Potential Biomarkers and Immunotherapeutic Targets for Cancers. Cancer Immunol. Res..

[193] Simpson A.J.G., Caballero O.L., Jungbluth A., Chen Y.-T., Old L.J. (2005). Cancer/testis antigens, gametogenesis and cancer. Nat. Rev. Cancer.

[194] Sang M., Lian Y., Zhou X., Shan B. (2011). MAGE-A family: Attractive targets for cancer immunotherapy. Vaccine.

[195] Wilgenhof S., Van Nuffel A., Corthals J., Heirman C., Tuyaerts S., Benteyn D., De Coninck A., Van Riet I., Verfaillie G., Vandeloo J. (2011). Therapeutic Vaccination with an Autologous mRNA Electroporated Dendritic Cell Vaccine in Patients with Advanced Melanoma. J. Immunother..

[196] Van Lint S., Renmans D., Broos K., Goethals L., Maenhout S., Benteyn D., Goyvaerts C., Du Four S., Van der Jeught K., Bialkowski L. (2015). Intratumoral Delivery of TriMix mRNA Results in T-cell Activation by Cross-Presenting Dendritic Cells. Cancer Immunol. Res..

[197] Van Nuffel A., Benteyn D., Wilgenhof S., Pierret L., Corthals J., Heirman C., van der Bruggen P., Coulie P.G., Neyns B., Thielemans K. (2012). Dendritic Cells Loaded with mRNA Encoding Full-length Tumor Antigens Prime CD4+ and CD8+ T Cells in Melanoma Patients. Mol. Ther..

[198] Boudewijns S., Bloemendal M., De Haas N., Westdorp H., Bol K.F., Schreibelt G., Aarntzen E.H.J.G., Lesterhuis W.J., Gorris M.A.J., Croockewit A. (2020). Autologous monocyte-derived DC vaccination combined with cisplatin in stage III and IV melanoma patients: A prospective, randomized phase 2 trial. Cancer Immunol. Immunother..

[199] De Vries I.J.M., Krooshoop D.J.E.B., Scharenborg N.M., Lesterhuis W.J., Diepstra J.H.S., Van Muijen G.N.P., Strijk S.P., Ruers T.J., Boerman O.C., Oyen W.J.G. (2003). Effective migration of antigen-pulsed dendritic cells to lymph nodes in melanoma patients is determined by their maturation state. Cancer Res..

[200] Morse M.A., Coleman R.E., Akabani G., Niehaus N., Coleman D., Lyerly H. (1999). Migration of human dendritic cells after injection in patients with metastatic malignancies. Cancer Res..

